# The causal relationship between blood cell indices and 28-day mortality in sepsis: a retrospective study and bidirectional Mendelian randomization analysis

**DOI:** 10.1186/s12879-024-09532-5

**Published:** 2024-06-22

**Authors:** Tao Zeng, Yina Sun, Shuru Chen, Jiahui Pang, Heping Wang, Xianghao Cai, Yingying Liao, Xiaolong Xiao, Yibo Zhang, Yutian Chong, Jiao Gong, Xinhua Li

**Affiliations:** 1 Department of Infectious Diseases, Key Laboratory of Liver Disease of Guangdong Province, The Third Affiliated Hospital of Sun Yat-Sen University, Guangzhou, 510630 China; 2 Department of Laboratory Medicine, The Third Affiliated Hospital of Sun Yat-Sen University, Guangzhou, 510630 China

**Keywords:** Blood cell indices, 28-day mortality in sepsis, Mendelian randomization analysis, Retrospective study, Platelet distribution width

## Abstract

**Background:**

Despite emerging evidence linking blood cell indices (BCIs) to sepsis mortality, the inconsistency of observational studies obscures the clarity of these associations. This study aims to clarify the causal influence of BCIs on 28-day mortality rates in sepsis patients.

**Methods:**

Utilizing univariable and multivariable Mendelian randomization (MR) analyses, we examined the impact of BCIs on sepsis mortality by analyzing data from extensive genome-wide association studies. The inverse-variance weighted (IVW) method was our primary analytic tool, complemented by several robustness checks to mitigate pleiotropy, including weighted median, mode-based estimates, MR-Egger regression, and MR-PRESSO. Subsequently, we conducted a retrospective study to further explore the correlation between platelet indices and 28-day mortality of sepsis using real-world data.

**Results:**

Our findings highlight a significant causal relationship between platelet distribution width (PDW) and 28-day mortality in sepsis, with the univariable Mendelian randomization approach yielding an odds ratio of 1.12 (95% CI, 1.06–1.26; *P* < 0.05). Multivariable analysis further substantiated PDW’s robust association with mortality risk (OR 1.23; 95% CI, 1.03–1.48; *P* < 0.05). Conversely, our analysis did not uncover significant correlations between the genetic predispositions to other BCIs—including red blood cell count, erythrocyte distribution width, platelet count, mean platelet volume, white blood cell count, neutrophil count, neutrophil percentage, lymphocyte count, and lymphocyte percentage—and 28-day mortality in sepsis. Additionally, an inverse MR analysis did not establish a causal impact of 28-day mortality in sepsis on PDW (OR 1.00; 95% CI, 1.00—1.07; *P* = 0.29). Moreover, a similar result was observed in the retrospective study.

**Conclusions:**

The study underscores the independent causal role of PDW in predicting 28-day mortality in sepsis, suggesting its potential utility in early patient assessment, risk stratification, and tailoring of therapeutic interventions.

**Supplementary Information:**

The online version contains supplementary material available at 10.1186/s12879-024-09532-5.

## Introduction

Sepsis is a complex and life-threatening infectious disease that results from a dysregulated host response to infection [1]. Annually, sepsis affects over 19 million people worldwide, resulting in over 6 million fatalities and a mortality rate exceeding one quarter [2, 3]. However, timely and effective treatment of sepsis is often hindered by the inability to quickly diagnose the condition. Studies have shown that a one-hour delay in effective antimicrobial and supportive therapy can result in a 5–10% increase in morbidity and mortality [4]. Biomarkers with high sensitivity and specificity are urgently needed to identify sepsis early and improve patient outcomes. Human blood cells (HBCs) are crucial in various physiological processes, including oxygen transport, coagulation, osmolarity regulation, and toxin clearance [5, 6]. Additionally, HBCs are involved in both innate and acquired immune responses, as well as inflammatory reactions within the body [7, 8]. Alterations in blood cell indices (BCIs) have been associated with various diseases, including tumors, immunodeficiencies, autoimmune diseases, and infections [9, 10].

Recent observational cohort studies have identified several common BCIs such as erythrocyte distribution width (RDW), platelet count (PLC), mean platelet volume (MPV), platelet distribution width (PDW), and monocyte distribution width (MDW), as potentially valuable tools for early sepsis screening and prognostic assessment [11–15]. However, the prognostic value of some BCIs in sepsis remains a subject of debate [16, 17]. Additionally, sepsis itself can have a wide range of effects on HBCs [18–20]. Therefore, the causal relationship between BCIs and sepsis risk remains unclear, limiting their value for new therapy development and sepsis pathophysiology exploration.

Genome-wide association studies (GWASs) are invaluable for revealing the genetic contributions to human diseases and providing novel insights into the intricate mechanisms governing disease etiology [21]. Mendelian randomization (MR) instrumental variable (IV) designs, by utilizing allelic randomization during meiosis and subsequent irreversible exposure to genotypes at conception, offer a diminished vulnerability to confounding or reverse causality when compared to traditional methods of observational analysis [22]. Here, we use data from large GWASs of sepsis patients to conduct an MR investigation to comprehensively explore whether circulating BCIs are likely to be causal pathways for the 28-day mortality of sepsis.

## Materials and methods

### Study design

**Fig. 1:**
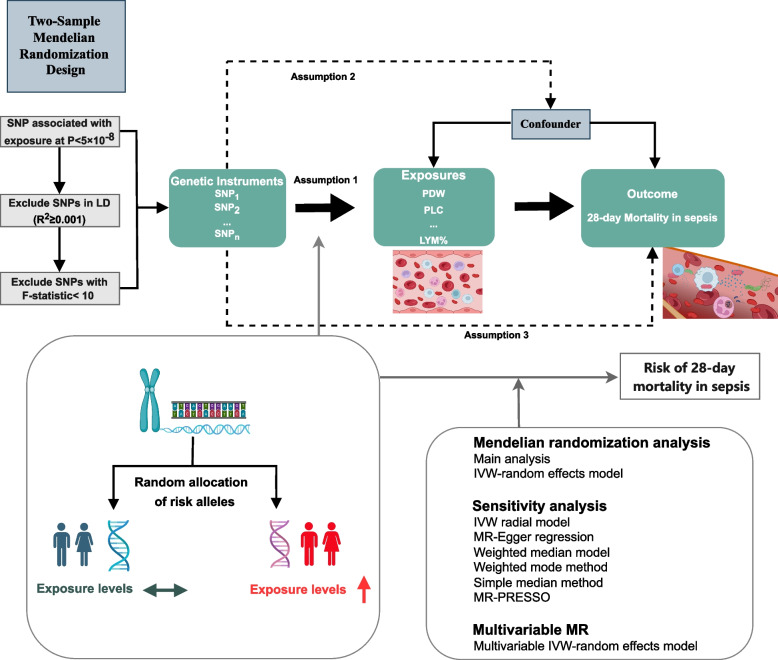
The overview design and assumptions of the MR design. IVW, inverse variance weighted; LD, linkage disequilibrium; LYM, lymphocyte cell count; LYM%, lymphocyte cell count percentage; MR, Mendelian randomization; MPV, Mean platelet (thrombocyte) volume; NEUT, Neutrophil; NEUT%, Neutrophil percentage; SNP, single nucleotide polymorphisms; PDW, Platelet distribution width; PLC, Platelet count; PRESSO, Pleiotropy Residual Sum and Outlier; RBC, Red blood cell (erythrocyte) count; RDW, Red blood cell (erythrocyte) distribution width; WBC, White blood cell (leukocyte) count

**Fig. 2:**
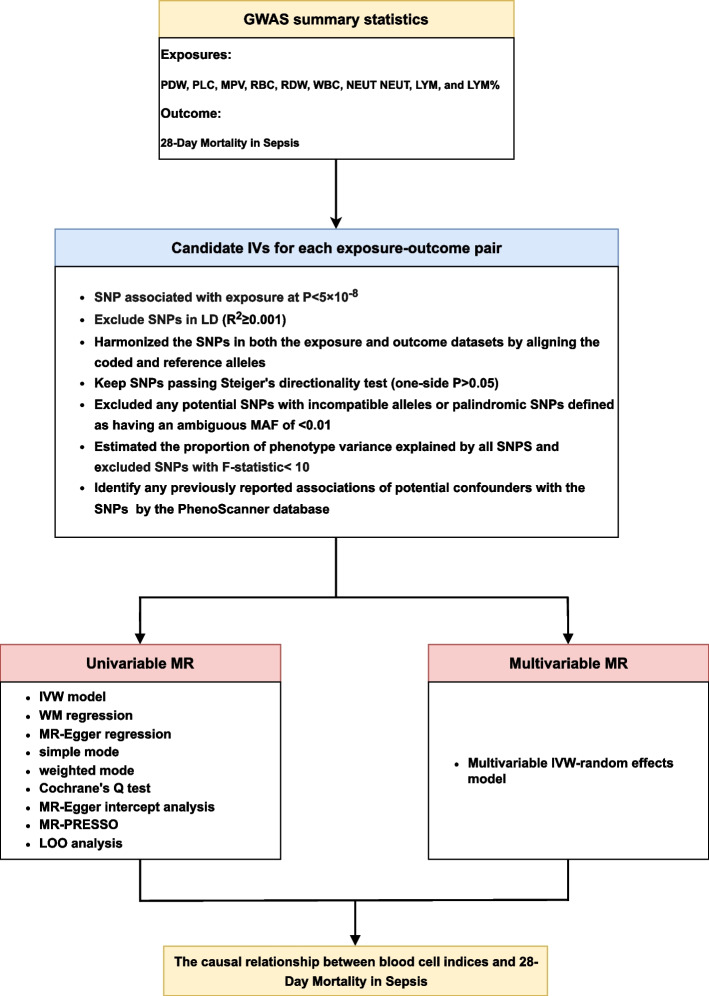
The flow chart illustrates the inclusion and exclusion of candidate SNPs for each exposure-outcome pair. GWAS, genome‑wide association studies; IVW, inverse variance weighted; LD, linkage disequilibrium; LYM, lymphocyte cell count; LYM%, lymphocyte cell count percentage; MAF, minor allele frequency; MR, Mendelian randomization; MPV, Mean platelet (thrombocyte) volume; NEUT, Neutrophil; NEUT%, Neutrophil percentage; SNP, single nucleotide polymorphisms; PDW, Platelet distribution width; PLC, Platelet count; PRESSO, Pleiotropy Residual Sum and Outlier; RBC, Red blood cell (erythrocyte) count; RDW, Red blood cell (erythrocyte) distribution width; WBC, White blood cell (leukocyte) count

Leveraging a two-sample MR framework, we first performed a univariable MR (UVMR) to test the association between genetic IVs respectively, as a proxy for the exposure, and the outcome. To ensure that the direct effect of each BCI (exposure) on mortality in sepsis (outcome) was not mediated by other factors, we further performed a multivariable MR (MVMR) analysis. Additionally, this MR design has to fulfill the following basic assumptions [23]: (i) IVs must be closely related to exposure; (ii) IVs are independent of potential confounding factors; (iii) The outcome is influenced by IVs only via the risk factors (Fig. 1). As the data employed in this study were publicly available, anonymized, and de-identified, it was exempt from ethical review authority approval. Figure 2 shows a flow diagram illustrating the study design and the steps of the MR analysis.

### BCIs data availability

The genetic IVs for the exposures in this study were acquired from published GWASs using summary data, sourced from the IEU Open GWAS Project (https://gwas.mrcieu.ac.uk/). We selected SNPs that demonstrated a significant association with BCIs at the stringent genome-wide significance threshold (*P* < 5 × 10^–8^) (Fig. 1). Specifically, for erythrocyte indices, two sets of instruments (SNPs for red blood cell (erythrocyte) count (RBC), and RDW) were employed for validation. For platelet indices, three sets of instruments (SNPs for PLC, PDW, and MPV) were employed for validation. For leukocyte indices, five sets of instruments (SNPs for white blood cell (leukocyte) count (WBC), neutrophil count (NEUT), neutrophil percentage (NEUT%), lymphocyte count (LYM), and lymphocyte percentage (LYM%)) were employed for validation. Notably, all cases and controls in these studies were selected from individuals of European descent, to reduce ethnic genetic differences as well as to reduce the effect of confounding factors. The available GWAS summary-level data for each trait are listed in Table 1.

**Table 1:** Summary of genome-wide association studies (GWAS) datasets in the study

GWAS ID	Trait	Author(Year)	Sample size	Number of SNPs	Population
ukb-d-30110_irnt	PDW	Neale lab(2018)	350,470	13,586,285	European
ebi-a-GCST004616	PDW	Astle WJ(2016)	164,433	29,144,965	European
ukb-d-30080_irnt	PLC	Neale lab(2018)	350,474	13,586,288	European
ukb-d-30100_irnt	MPV	Neale lab(2018)	350,470	13,586,285	European
ukb-d-30010_irnt	RBC	Neale lab(2018)	350,475	13,586,289	European
ukb-d-30070_irnt	RDW	Neale lab(2018)	350,473	13,586,288	European
ukb-d-30000_irnt	WBC	Neale lab(2018)	350,470	13,586,282	European
ukb-d-30200_irnt	NEUT%	Neale lab(2018)	349,861	13,586,283	European
ukb-d-30140_irnt	NEUT	Neale lab(2018)	349,856	13,586,292	European
ukb-d-30180_irnt	LYM%	Neale lab(2018)	349,861	13,586,283	European
ukb-d-30120_irnt	LYM	Neale lab(2018)	349,856	13,586,292	European
ieu-b-5086	Sepsis^a^	Hamilton F (2021)	486,484	12,243,487	European

*Abbreviations: PDW* Platelet distribution width, *PLC* Platelet count, *MPV* Mean platelet (thrombocyte) volume, *RBC* Red blood cell (erythrocyte) count, *RDW* Red blood cell (erythrocyte) distribution width, *WBC* White blood cell (leukocyte) count, *NEUT%* Neutrophil percentage, *LYM* Lymphocyte cell count, *LYM%* Lymphocyte cell count percentage

Sepsis^a^, 28-day death in sepsis

### Genetic instrument selection

All IVs were clumped within a window of 10 megabase pairs (Mb) using a strict linkage disequilibrium (LD) threshold of (*R*^2^ = 0.001) to determine that the SNPs were independent. MR-Steiger analysis was utilized to assess the direction of the potential causal association between the extracted SNPs related to the risk factors and 28-day mortality in sepsis [24]. Subsequently, we harmonized the SNPs in both the exposure and outcome datasets by aligning the coded and reference alleles and excluded any potential SNPs with incompatible alleles or palindromic SNPs, which were defined as having an ambiguous minor allele frequency of < 0.01 [25]. Given that missing SNPs had a negligible effect on the results, we used only the available SNPs for all traits as IVs and did not replace any missing SNPs with proxies in the outcome data [26]. We estimated the proportion of phenotype variance explained by all SNPs and assessed their strength using the F-statistic (beta^2^/se^2^) and a threshold of *F* > 10 was considered sufficient, aligning with the first MR assumption and avoiding bias towards weak IVs [27]. To meet the second and third criteria for MR, we used the PhenoScanner database (http://www.phenoscanner.medschl.cam.ac.uk/) to identify any previously reported associations of potential confounders with the SNPs used as instruments.

### Data sources for the outcome

We obtained summary-level genetic data for GWASs on 28-day mortality in sepsis from the IEU Open GWAS Project (ID: ieu-b-5086) (https://gwas.mrcieu.ac.uk/datasets/ieu-b-5086/), which were analyzed using Regenie v2.2.4, with adjustments made for age, sex, chip type, and the first 10 principal components. In total, the study had 486,484 sample sizes from the European population, including 1,896 cases and 484,588 controls, for a total of 12,243,487 SNPs (Table 1).

#### Retrospective study

**Fig. 3:**
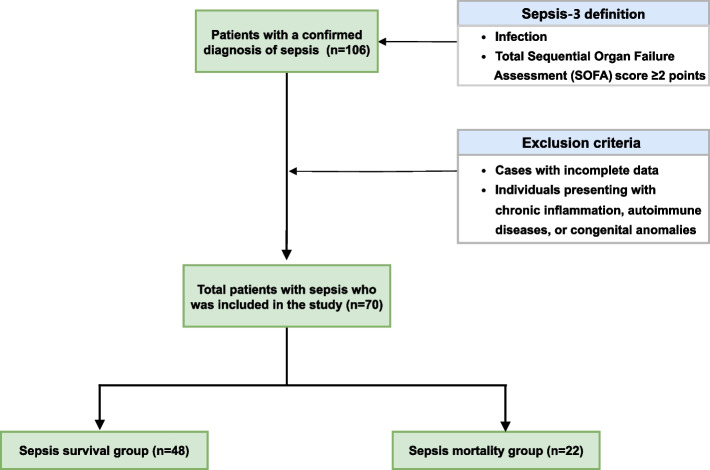
Flowchart of participant selection

This retrospective study utilized historical medical records from patients admitted to the Department of Infectious Diseases at the Third Affiliated Hospital of Sun Yat-Sen University in Guangzhou, China. The criteria for sepsis diagnosis adhered to the Sepsis-3 definition, characterized by an acute increase in the total Sequential Organ Failure Assessment (SOFA) score ≥ 2 points consequent to an infection. Data were collected from all subjects with a confirmed diagnosis of sepsis from January 2023 to May 2024. Data within 24 h of their admission were obtained from the hospital’s laboratory information system, including platelet indices such as PLC, PDW, and MPV, as well as patient age, sex, and diagnosis. Exclusion criteria encompassed cases with incomplete data, as well as patients presenting with chronic inflammation, autoimmune conditions, or congenital anomalies—disorders present at birth due to genetic factors or maternal exposure to teratogens, including congenital cardiovascular malformations, inherited hematologic conditions such as thalassemia and hereditary spherocytosis, and metabolic disorders of genetic origin. These individuals were omitted to preclude potential biases associated with aberrant hematological profiles, hepatic function, and inflammatory markers. A total of 106 individuals were initially included. After applying the exclusion criteria, 70 patients with sepsis were included in the study, of whom 48 survived and 22 died (Fig. 3). The use of retrospective clinical data from patients has been approved by the Ethics Committee of the Third Affiliated Hospital of Sun Yat-sen University and the study was conducted in accordance with the principles outlined in the Declaration of Helsinki.

### Statistical analysis

Heterogeneity among the SNPs was assessed by calculating Cochrane's Q statistic. When the *P*-value from Cochrane's Q test was less than 0.05, indicating the presence of heterogeneity, a random-effect inverse-variance weighted (IVW) model was employed as the primary analysis method [28].However, to address the robustness of our IVW estimate, we also performed a sensitivity analysis using complementary methods based on MR-Egger and weighted median (WM) regression, which make different assumptions about the validity of the genetic variants as IVs [28–30]. More specifically, the WM method, which necessitates a minimum of 50% weight attributed to valid IVs, was additionally employed to estimate the causal effects [31]. We used the MR-Egger regression, which gives consistent estimates even when all genetic variants are invalid IVs, to evaluate possible directional pleiotropy [30]. In addition, we also applied the simple mode and the weighted mode methods to infer causal relationships. Furthermore, we used the MR-PRESSO framework to identify and correct any horizontal pleiotropic outliers that could bias the IVW estimate by using the outlier removal kit [32]. The distortion test shows a significant change in the estimate before and after the outlier correction if the *P*-value < 0.05. In addition, we performed the leave-one-out analysis to examine whether a single outlier variant influenced the effect estimates and to identify any high influence points [33].

We used the SNPs in each of the GWASs that met our UVMR selection criteria to construct instruments. To correct for both measured and unmeasured pleiotropy, we used the MVMR extension of the IVW MR method [34, 35]. We extracted the effects and standard errors of the SNPs from the exposure and outcome GWAS. The MR estimates are reported as odds ratios (ORs) and 95% confidence intervals (CIs). Data were presented as “mean ± standard deviation (SD),” “median (quartile 1 (Q1), Q3),” or “frequency (percentage)” as appropriate. Quantitative variables underwent comparative analysis via the Student’s t-test or the Mann–Whitney U-test, while categorical variables were. We used R software (version 4.2.2) and the TwoSampleMR (version 0.5.7) and MR-PRESSO (version 1.0) packages for all analyses.

## Results

### Selection of genetic IVs of exposures

In total, there were 570 independent IVs (SNPs) for PDW, 719 SNPs for PLC, 785 SNPs for MPV, 554 SNPs for RBC, 16 SNPs for RDW, 465 SNPs for WBC, 384 SNPs for NEUT, 349 SNPs for NEUT%, 489 SNPs for LYM, and 395 SNPs for LYM%, respectively (Fig. 3). There are no strong associations between the outcome and the selected IVs, which suggests their specific relevance to 28-day mortality in sepsis.

### Univariable Mendelian randomization

#### Causal effect of BCIs on 28-day mortality in sepsis

**Fig. 4:**
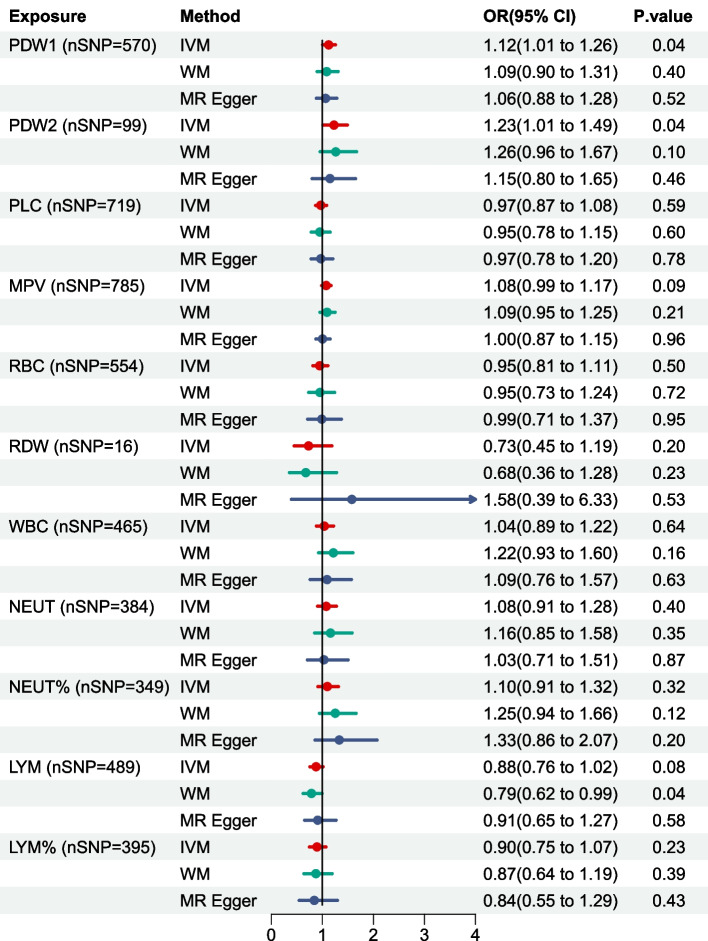
UVMR Results of BCIs on Risk of 28-day Mortality in sepsis. Results from 2-sample UVMR analysis; estimated associations reported as OR of outcome per unit increase in log odds of 28-day mortality in sepsis. All relevant SNPs were identified in a GWAS as having reached a selection threshold of *P* < 0.05 × 10^–8^ and pruned at linkage disequilibrium *R*^2^ < 0.001. IVW, inverse variance weighted; LYM, lymphocyte cell count; LYM%, lymphocyte cell count percentage; MPV, Mean platelet (thrombocyte) volume; NEUT, Neutrophil; NEUT%, Neutrophil percentage; N SNP, number of single nucleotide polymorphisms; OR, odds ratio; PDW, Platelet distribution width; PDW1, Data from GWAS ID ukb-d-30110_irnt; PDW2, Data from GWAS ID ebi-a-GCST004616; PLC, Platelet count; RBC, Red blood cell (erythrocyte) count; RDW, Red blood cell (erythrocyte) distribution width; WBC, White blood cell (leukocyte) count; WM, weighted median; UVMR, Univariable Mendelian randomization

Conventional UVMR analysis suggested that only genetically determined PDW had a statistically significant causal effect on 28-day mortality in sepsis, with an OR of 1.12 (95% CI, 1.06–1.26; *P* < 0.05) using the IVW method (Fig. 4). The scatter plots for effect sizes of SNPs for PDW are shown in Figure S1A. In contrast, the other four methods, including WM (OR, 1.09; 95% CI, 0.90–1.31; *P* = 0.40), MR Egger (OR, 1.06; 95% CI, 0.88–1.28; *P* = 0.52), simple mode (OR, 1.04; 95% CI, 1.01–1.26; *P* = 0.86), and weighted mode (OR, 1.04; 95% CI, 0.83–1.31; *P* = 0.72), yielded similar but non-significant estimates (Table S3). The IVs of PDW used in these analyses are detailed in Table S2. As for the SNPs of PDW, Cochrane's Q test indicated the absence of heterogeneity (Cochrane’s Q = 614.7; *P* = 0.09), and the MR-Egger intercept analysis did not provide evidence of horizontal pleiotropy (intercept = 0.002, *P* = 0.475). Furthermore, as shown in Figure S1B, the funnel plot is symmetrical, suggesting that there is no substantial publication bias or other major sources of bias affecting the results. Conventional IVW, leave-one-out showed that all of the SNPs were on the right side of “0”, indicating that removing any SNP would not have a large impact on the causal correlation estimation, and the MR analysis results were robust. Nevertheless, there were no significant associations observed between genetic liability to other BCIs (all *P* > 0.05), such as RBC, RDW, WBC, NEUT, NEUT%, LYM, LYM%, PLC, and MPV, with 28-day mortality in sepsis. Furthermore, we validated these results using an independent dataset (GWAS ID, ebi-a-GCST004616). In this additional analysis, employing the IVW method with 99 SNPs, we also observed a significant association between PDW and 28-day mortality in sepsis, with an OR of 1.23 (95% CI, 1.01–1.49, *P* < 0.05). These findings supported the hypothesis that higher PDW increases the risk of death in sepsis patients.

#### Casual effect of 28-day mortality in sepsis on PDW

**Fig. 5:**
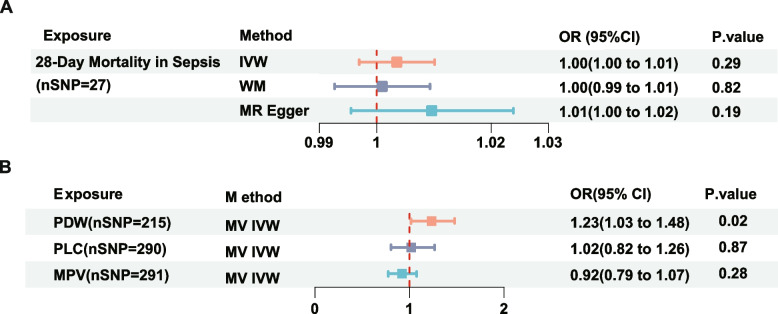
Inverse Mendelian Randomization analysis and multivariable Mendelian randomization results. **A** Inverse MR results of the effect of 28-day mortality in sepsis on PDW. **B** MVMR results of PDW, PLC, and MPV on Risk of 28-day mortality in sepsis. Abbreviations: MPV, Mean platelet (thrombocyte) volume; MVMR, multivariable Mendelian randomization; MV IVW, multivariable inverse variance weighted; OR, odds ratio; PDW, Platelet distribution width; PLC, Platelet count

Given that sepsis itself can also influence alterations in platelets, we subsequently conducted a reverse MR study to corroborate the impact of PDW on sepsis. Considering the genetic predisposition to 28-day mortality in sepsis as the exposure variable, the outcomes of the reverse MR analyses are presented in Supplementary Table S4. The random-effects IVW methodology yielded no substantial evidence supporting a causal relationship between 28-day mortality in sepsis and PDW (OR, 1.00; 95% CI, 1.00–1.07; *P* = 0.29), as shown in Fig. 5A. Similar findings were obtained with MR-Egger regression, weighted median, and mode analyses, revealing no evidence of a causal relationship between 28-day sepsis mortality and PDW (Table S4).

### Multivariable Mendelian randomization

To further evaluate the significance of PDW as a risk factor for 28-day mortality in sepsis, we performed an additional MVMR analysis following our UVMR analysis to assess the impact of major confounders and determine the independence of causal effects. we extracted 215 SNPs for PDW, 290 SNPs for PLC, and 291 SNPs for MPV. In MVMR, assessing the genetic liabilities for PDW, PLC, and MPV jointly, PDW retained a robust relationship with 28-day mortality in sepsis, and the effect size of association was amplified with an OR of 1.23 (95% CI, 1.03–1.48; *P* < 0.05) using the IVW method. However, consistent with the findings from the previous UVMR analysis, the IVW analysis did not yield statistically significant results for PLC (OR, 1.02; 95% CI, 0.82–1.26; *P* = 0.87) and MPV (OR, 0.92; 95% CI, 0.79–1.07; *P* = 0.28) (Fig. 5B). The above findings further support a causal relationship between PDW and 28-day mortality in sepsis.

### Results of the retrospective study

**Fig. 6:**
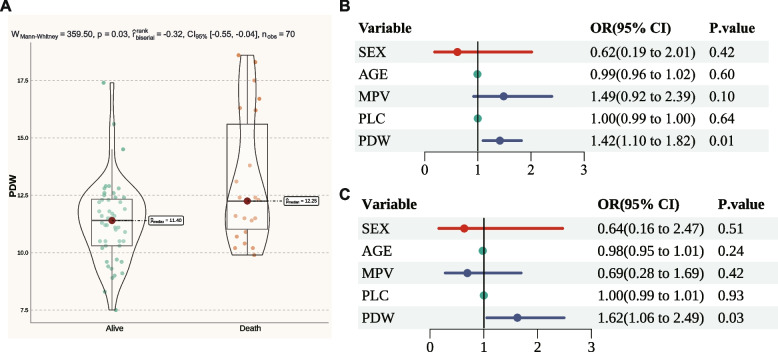
Results of the retrospective study. **A** Comparison of PDW levels between the sepsis mortality and sepsis survival groups. **B** Univariate logistic regression analysis results. **B** multivariable logistic regression analysis results. Abbreviations: MPV, Mean platelet (thrombocyte) volume; OR, odds ratio; PDW, Platelet distribution width; PLC, Platelet count

The baseline characteristics for patients with sepsis mortality group and sepsis survival groups are shown in Table 2. In our analysis, it was found that PDW levels were notably elevated in the sepsis mortality group compared to the sepsis survival groups (Fig. 6A). Furthermore, while MPV levels were also increased in the sepsis mortality group, this difference did not reach statistical significance, possibly due to the limited sample size. Subsequently, a univariate logistic regression model was employed to investigate the relationship between platelet indices and mortality from sepsis. The results indicated that PDW was a risk factor for higher mortality in patients with sepsis, with an OR of 1.42 (95% CI = 1.10 to 1.82, *p* = 0.01), as illustrated in Fig. 6B. Subsequently, following the multivariable regression analysis, PDW remained a risk factor for sepsis mortality. This finding further supports the association between PDW and sepsis mortality, further supporting the association between PDW and sepsis mortality (Fig. 6C).

**Table 2:** Demographic characteristics and platelet indices of the included patients

Variables	Sepsis			*P* value
	Total (*n* = 70)	Mortality group (*n* = 48)	Survival group (*n* = 22)	
**MPV (FL)**	10.61 ± 1.1	10.46 ± 1.01	10.93 ± 1.23	0.130
**PLC (10**^**9**^**/L)**	136 (91.25, 219)	137 (110.25, 219)	115 (34.75, 224.75)	0.327
**Gender**				0.532
** Male**	15 (21%)	9 (19%)	6 (27%)	
** Female**	55 (79%)	39 (81%)	16 (73%)	
**Age(year)**	58.43 ± 17.74	59.19 ± 17.41	56.77 ± 18.75	0.612
**PDW (%)**	11.55 (10.5, 12.47)	11.4 (10.3, 12.33)	12.25 (11.03, 15.6)	0.033

*Abbreviations: FL* Femtoliter, *MPV* Mean platelet volume, *PLC* Platelet count, *PDW* Platelet distribution width

## Discussion

BCIs have been recognized for their diagnostic, pathophysiological, and prognostic value in sepsis, but their causal role, and if so, their causal direction, remain uncertain. This study aimed to explore the causal relationships between BCIs and 28-day mortality in sepsis by conducting an MR analysis. MR analysis, as utilized in our study, is a research method that employs genetic variants as instrumental variables to assess causality [22]. Based on Mendel's laws, it assumes random allocation of genes, thus mitigating many confounding factors and issues of reverse causality common in traditional epidemiological studies [22]. While strikingly, we found a remarkable and consistent genetic association between PDW and 28 day mortality in sepsis risk in both UVMR and MVMR models. Subsequently, retrospective analysis data similarly affirmed that elevated PDW serves as a risk factor for mortality in sepsis. However, no significant associations of genetic liability to other BCIs were observed, which is inconsistent with previous findings [11–15].

Traditionally, platelets are typically known for their role in hemostasis; however, recent evidence highlights their significant involvement in sepsis, inflammation, and immune responses [6, 20]. Various cohort studies have emphasized the importance of platelet parameters such as PLC, PDW, and MPV in the early diagnosis and prognosis of sepsis [12, 13, 36–38], although some studies question their prognostic value [16, 39]. In line with prior research, our MR study provided evidence that a genetic predisposition to higher PDW is independently and causally associated with an increased risk of 28-day mortality in sepsis (IVW: OR, 1.12; 95% CI, 1.06–1.26; *P* < 0.05). However, unlike previous studies, we found no obvious evidence that genetic predisposition to PLC and MPV is causally associated with the risk of mortality in sepsis.

Nevertheless, a larger question is how PDW affects the prognosis of sepsis, and why MPV, another indicator of platelet size, has no relationship with sepsis mortality. PDW serves as an indicator of the morphological variability within platelets and is potentially linked to their functional status and production rate [40]. Therefore, an elevated PDW may reflect the activated state of platelets. In the context of sepsis, platelets experience heightened activation and aggregation, subsequently culminating in the formation of microvascular thrombosis [41]. Consequently, heightened PDW levels may signify an augmented risk of platelet aggregation and thrombus development, thereby contributing to compromised microcirculation, organ hypoperfusion, and multi-organ dysfunction [41, 42]. In contrast, MPV only reflects the average volume and may not capture the variability in their functional activity.

On the other hand, increased PDW may also be related to the inflammatory state of sepsis patients. Activated platelets are capable of releasing a large number of inflammatory mediators, thereby modulating the response of leukocytes and endothelial cells to inflammatory stimuli [43]. When platelets are activated, they can secrete a plethora of chemotactic factors, cytokines, and adhesion molecules, such as IL-1, CXCL1, and PF4 [44]. Among them, IL-1 acts as a central mediator in the cascade of cytokine reactions, further inducing vascular smooth muscle cells to release IL-6 and IL-8, as well as endothelial cells to release CCL2 [45, 46]. The ensuing inflammatory reactions can precipitate a cascade of events including a cytokine storm, endothelial cell injury, release of tissue factors, and activation of the coagulation cascade, ultimately leading to the development of disseminated intravascular coagulation (DIC), propensity for bleeding, and shock [6, 42]. Moreover, platelets can also form aggregates with leukocytes, exerting further influence on leukocyte functionality [43]. Platelet-leukocyte interactions can further enhance the adhesion characteristics and phagocytic activity of neutrophils, inducing an inflammatory response, which is crucial in the innate immune system for combating microbial infections [47]. However, in sepsis, there is excessive activation of the innate immune system, particularly neutrophil hyperactivation, which is associated with tissue damage, multi-organ failure, DIC, and other microvascular pathologies [48]. Furthermore, abundant evidence has shown that pathogens such as *Staphylococcus aureus*, *Streptococcus pneumoniae*, and *Escherichia coli* can bind to platelets and further activate platelet function [49], which may play a crucial role in the pathogenesis of sepsis.

As for the leukocyte and erythrocyte indices, although previous studies have indicated that most of them, such as RDW, are associated with the prognosis of sepsis, our MR analysis did not find any significant causal correlation. Furthermore, our study also provided new insights into some controversial issues. For example, RDW has been considered a valid prognostic marker in most studies, but some studies have challenged its importance [16, 17, 39]. Our study found that RDW had no clear causal association with sepsis severity (Fig. 3). In summary, to elucidate the intricate interplay between these hematological indices and sepsis, future research endeavors should focus on unraveling the underlying molecular pathways and biological mechanisms involved.

### Strengths and limitations

We used summary genetic associations from the largest GWASs available, which are crucial for identifying small effect sizes. Moreover, the utilization of the MVMR model represents a significant strength. MVMR enables the incorporation of multiple genetic variants, accounting for their effects on both the exposure and outcome variables. Additionally, we conducted a single-center retrospective analysis to further validate the MR results. To our knowledge, this is the first study using two-sample MR analysis to investigate the causal relationship between BCIs and mortality risk in sepsis.

However, the results should be interpreted cautiously due to the following limitations. Despite the large sample size used in our analysis, we acknowledge the possibility of overlooking weak associations, especially those involving exposures composed of some SNPs that can explain small phenotypic variations. In addition, it is important to recognize the potential for bias resulting from horizontal pleiotropy. we found consistent results across various sensitivity analyses, and limited evidence of significant pleiotropic effects was observed in MR-Egger and MR-PRESSO analyses. Thirdly, the lack of detailed clinical data at a large scale prevented us from performing subgroup analyses, which could introduce potential biases. Fourth, the population restriction in our study may constrain the applicability of our findings to other populations.

## Conclusions

We provide preliminary genetic evidence that PDW is a causal risk factor for increased 28-day mortality risk in sepsis, which may facilitate early identification of critically ill patients in clinical settings. Subsequently, retrospective analysis data similarly affirmed that elevated PDW serves as a risk factor for mortality in sepsis. Furthermore, we find no evidence of causal effects for some previously predictive BCIs such as RDW and PLC, and offer new insights into some controversial indicators. In future studies, identifying comprehensive risk factors for sepsis and developing novel prediction models for sepsis can help to stratify individuals at specific risk and provide decision support for personalized interventions.

### Supplementary Information

Supplementary Material 1.

## Data Availability

The genetic IVs for the exposures in this study were acquired from published GWASs using summary data, sourced from the IEU open GWAS project (https://gwas.mrcieu.ac.uk/). We selected SNPs that demonstrated a significant association with BCIs at the stringent genome-wide significance threshold (P < 5 × 10–8).

## Abbreviations

BCIs: Blood cell indices
CCL2: Chemokine ligand 2
CI: Confidence intervals
CXCL1: Recombinant human Chemokine Ligand 1
CXCL5: Recombinant human Chemokine Ligand 5
CXCL7: Recombinant human Chemokine Ligand 7
DIC: Disseminated intravascular coagulation
GWAS: Genome-wide association studies
HBCs: Human blood cells
IEU: International English non-academic exchanges Union
IV: Instrumental variable
IVW: Inverse-variance weighting
LD: Linkage disequilibrium
LYM: Lymphocyte count
LYM%: Lymphocyte percentage
MB: Megabase pairs
MDW: Monocyte distribution width
MPV: Mean platelet volume
MR: Mendelian randomization
MR-PRESSO: Mendelian randomization pleiotropy residual sum and outlier
MVMR: Multivariate mendelian randomization
NEUT: Neutrophil count
NEUT%: Neutrophil percentage
OR: Odds ratios
P: Probability
PDW: Platelet distribution width
PF4: Platelet Factor-4
PLC: Platelet count
RDW: Red cell distribution width
SNPs: Single nucleotide polymorphisms
SOFA: Sequential Organ Failure Assessment
UVMR: Univariable mendelian randomization
WBC: White blood cell count
WM: Weighted median
