# Supplementary material for: The causal relationship between blood cell indices and 28-day mortality in sepsis: a retrospective study and bidirectional Mendelian randomization analysis
Source: BMC Infect Dis. 2024 Jun 22;24:619. doi: 10.1186/s12879-024-09532-5 (PMC11193192; doi:10.1186/s12879-024-09532-5)
Supplement: Supplementary file 1 — Supplementary Material 1. [file 12879_2024_9532_MOESM1_ESM.docx]

**Supplementary materials**

**Table S1.** **Summary of genome-wide association studies (GWAS) datasets in the study**

| **GWAS ID** | **Trait** | **Author（Year）** | **Sample size** | **Number of SNPs** | **Category** | **Population** | **Download** |
| --- | --- | --- | --- | --- | --- | --- | --- |
| ukb-d-30110_irnt | PDW | Neale lab（2018） | 350,470 | 13,586,285 | Continuous | European | https://gwas.mrcieu.ac.uk/datasets/ukb-d-30110_irnt/ |
| ebi-a-GCST004616 | PDW | Astle WJ（2016） | 164,433 | 29,144,965 | NA | European | https://gwas.mrcieu.ac.uk/datasets/ebi-a-GCST004616/ |
| ukb-d-30080_irnt | PLC | Neale lab（2018） | 350,474 | 13,586,288 | Continuous | European | https://gwas.mrcieu.ac.uk/datasets/ukb-d-30080_irnt/ |
| ukb-d-30100_irnt | MPV | Neale lab（2018） | 350,470 | 13,586,285 | Continuous | European | https://gwas.mrcieu.ac.uk/datasets/ukb-d-30100_irnt/ |
| ukb-d-30010_irnt | RBC | Neale lab（2018） | 350,475 | 13,586,289 | Continuous | European | https://gwas.mrcieu.ac.uk/datasets/ukb-d-30010_irnt/ |
| ukb-d-30070_irnt | RDW | Neale lab（2018） | 350,473 | 13,586,288 | Continuous | European | https://gwas.mrcieu.ac.uk/datasets/ukb-d-30070_irnt/ |
| ukb-d-30000_irnt | WBC | Neale lab（2018） | 350,470 | 13,586,282 | Continuous | European | https://gwas.mrcieu.ac.uk/datasets/ukb-d-30000_irnt/ |
| ukb-d-30200_irnt | NEUT% | Neale lab（2018） | 349,861 | 13,586,283 | Continuous | European | https://gwas.mrcieu.ac.uk/datasets/ukb-d-30200_irnt/ |
| ukb-d-30140_irnt | NEUT | Neale lab（2018） | 349,856 | 13,586,292 | Continuous | European | https://gwas.mrcieu.ac.uk/datasets/ukb-d-30140_irnt/ |
| ukb-d-30180_irnt | LYM% | Neale lab（2018） | 349,861 | 13,586,283 | Continuous | European | https://gwas.mrcieu.ac.uk/datasets/ukb-d-30180_irnt/ |
| ukb-d-30120_irnt | LYM | Neale lab（2018） | 349,856 | 13,586,292 | Continuous | European | https://gwas.mrcieu.ac.uk/datasets/ukb-d-30120_irnt/ |
| ieu-b-5086 | Sepsis* | Hamilton F (2021) | 486,484 | 12,243,487 | NA | European | https://gwas.mrcieu.ac.uk/datasets/ieu-b-5086/ |

**Abbreviations:** PDW, Platelet distribution width；PLC, Platelet count； MPV, Mean platelet (thrombocyte) volume； RBC, Red blood cell (erythrocyte) count； RDW, Red blood cell (erythrocyte) distribution width； WBC, White blood cell (leukocyte) count； NEUT%, Neutrophil percentage； LYM, lymphocyte cell count； LYM%, lymphocyte cell count percentage. Sepsis*, 28-day death in sepsis.

**Table S2.** **SNPs as instrumental variables in the study of the effect of PDW on 28-day mortality in sepsis risk**

| **Exposure** | **SNP** | **Effect allele** | **Other allele** | **Exposure** | | | | **Outcome** | | | |
| --- | --- | --- | --- | --- | --- | --- | --- | --- | --- | --- | --- |
|  |  |  |  | **Beta** | **SE** | **P value** | **Eaf** | **Beta** | **SE** | **P value** | **Eaf** |
| PDW (id:ukb-d-30110_irnt) | rs10036404 | G | C | -0.0460561 | 0.00805174 | 1.07E-08 | 0.0225332 | -0.0706106 | 0.112069 | 0.528655 | 0.0232364 |
|  | rs1006409 | G | A | 0.0331709 | 0.00236959 | 1.63E-44 | 0.460011 | 0.0433855 | 0.033452 | 0.194648 | 0.459243 |
|  | rs10109515 | C | T | -0.0272203 | 0.00293921 | 2.03E-20 | 0.794874 | -0.0791408 | 0.0414258 | 0.056079 | 0.794124 |
|  | rs10122534 | A | G | 0.0184696 | 0.00271763 | 1.08E-11 | 0.256972 | 0.0147919 | 0.0383569 | 0.699765 | 0.257697 |
|  | rs10147793 | T | A | -0.0233336 | 0.00420102 | 2.79E-08 | 0.0867093 | 0.0100277 | 0.0586836 | 0.86432 | 0.0886283 |
|  | rs10179126 | G | C | -0.0146154 | 0.00245696 | 2.71E-09 | 0.365147 | -0.062966 | 0.0347398 | 0.0699085 | 0.364234 |
|  | rs102274 | C | T | 0.0172384 | 0.00248285 | 3.85E-12 | 0.34665 | 0.074509 | 0.0350921 | 0.0337334 | 0.345322 |
|  | rs10234379 | G | A | -0.0260827 | 0.00404821 | 1.17E-10 | 0.0948586 | 0.0252516 | 0.0570934 | 0.658282 | 0.0954536 |
|  | rs1034564 | T | C | -0.0214026 | 0.00261158 | 2.51E-16 | 0.285655 | 0.00695139 | 0.0368688 | 0.85045 | 0.285435 |
|  | rs1036332 | C | A | 0.0181976 | 0.00269951 | 1.57E-11 | 0.739072 | -0.0118031 | 0.0381558 | 0.757063 | 0.73814 |
|  | rs10466710 | G | A | -0.0200831 | 0.00245265 | 2.66E-16 | 0.374244 | -0.0530194 | 0.0345768 | 0.125182 | 0.37416 |
|  | rs1060431 | A | G | -0.0877527 | 0.00455341 | 1.02E-82 | 0.0727305 | -0.00609529 | 0.063638 | 0.923695 | 0.0741637 |
|  | rs10736152 | C | G | -0.0159084 | 0.00237544 | 2.13E-11 | 0.512482 | -0.0123497 | 0.0335966 | 0.713181 | 0.509577 |
|  | rs10737805 | G | A | 0.0218982 | 0.00316372 | 4.47E-12 | 0.831375 | 0.0515911 | 0.0447272 | 0.24872 | 0.832228 |
|  | rs10774122 | C | T | -0.0406346 | 0.00241824 | 2.44E-63 | 0.604136 | 0.000708301 | 0.0341693 | 0.983462 | 0.60469 |
|  | rs10792832 | G | A | -0.0138135 | 0.00244413 | 1.59E-08 | 0.629069 | 0.0223794 | 0.0344711 | 0.516195 | 0.630035 |
|  | rs10800573 | G | A | 0.0191473 | 0.00254564 | 5.42E-14 | 0.316314 | -0.00933724 | 0.0358656 | 0.794602 | 0.318675 |
|  | rs10808546 | T | C | -0.0184245 | 0.00238466 | 1.11E-14 | 0.449286 | -0.0399553 | 0.033662 | 0.235246 | 0.446643 |
|  | rs10811664 | A | G | 0.0193448 | 0.00327186 | 3.37E-09 | 0.154915 | 0.00797606 | 0.0460451 | 0.862477 | 0.156787 |
|  | rs10849408 | G | A | 0.0268397 | 0.00247935 | 2.64E-27 | 0.633719 | 5.11E-05 | 0.0349814 | 0.998835 | 0.630709 |
|  | rs10849448 | G | A | 0.0175754 | 0.00280079 | 3.50E-10 | 0.753152 | 0.0109343 | 0.0394955 | 0.781896 | 0.752396 |
|  | rs10863358 | C | G | -0.0164218 | 0.00300012 | 4.41E-08 | 0.194741 | 0.0707837 | 0.0421049 | 0.0927385 | 0.197872 |
|  | rs10868070 | A | G | -0.0185718 | 0.00268603 | 4.71E-12 | 0.262635 | 0.0106212 | 0.037897 | 0.779275 | 0.263672 |
|  | rs10876550 | A | G | -0.0486365 | 0.00237479 | 3.66E-93 | 0.558979 | -0.0109854 | 0.0335383 | 0.743252 | 0.558802 |
|  | rs10878596 | A | G | -0.0157442 | 0.00241759 | 7.41E-11 | 0.604854 | 0.025933 | 0.0341382 | 0.447465 | 0.602991 |
|  | rs10886430 | G | A | 0.0379586 | 0.00361994 | 1.01E-25 | 0.126979 | 0.0202353 | 0.0510699 | 0.691938 | 0.127365 |
|  | rs10892563 | C | T | -0.0134138 | 0.00242887 | 3.34E-08 | 0.381417 | 0.0288855 | 0.0342839 | 0.399487 | 0.382207 |
|  | rs10898864 | T | C | 0.0151422 | 0.00265963 | 1.25E-08 | 0.269504 | 0.0236561 | 0.0375471 | 0.52867 | 0.268011 |
|  | rs10900450 | G | C | -0.0178995 | 0.00246072 | 3.49E-13 | 0.64094 | 0.003731 | 0.0346869 | 0.914343 | 0.639138 |
|  | rs10914053 | T | C | 0.144735 | 0.00311651 | 1.00E-200 | 0.825606 | 0.0176226 | 0.0441357 | 0.689684 | 0.826088 |
|  | rs10915874 | T | C | 0.023932 | 0.00372573 | 1.33E-10 | 0.113847 | 0.0735707 | 0.0524397 | 0.160629 | 0.113742 |
|  | rs10916498 | C | G | 0.0364398 | 0.00621848 | 4.64E-09 | 0.0380603 | -0.0455644 | 0.0876597 | 0.603212 | 0.0380962 |
|  | rs10940286 | A | G | 0.0148272 | 0.00253676 | 5.07E-09 | 0.682027 | 0.0574357 | 0.0358568 | 0.109198 | 0.682142 |
|  | rs10947589 | A | G | 0.0142673 | 0.00258944 | 3.59E-08 | 0.299815 | -0.0766359 | 0.0364535 | 0.0355279 | 0.298242 |
|  | rs10974576 | T | G | -0.014799 | 0.00253779 | 5.50E-09 | 0.683852 | 0.0238663 | 0.0357407 | 0.504286 | 0.682448 |
|  | rs11013176 | G | A | -0.0232061 | 0.00264257 | 1.62E-18 | 0.280894 | -0.106004 | 0.0373274 | 0.00451357 | 0.282023 |
|  | rs11014291 | C | T | 0.0154772 | 0.00244621 | 2.50E-10 | 0.372239 | 0.0779783 | 0.0345818 | 0.0241402 | 0.368581 |
|  | rs11052214 | T | C | -0.0313744 | 0.00342196 | 4.82E-20 | 0.138404 | -0.0392524 | 0.0481836 | 0.415277 | 0.138776 |
|  | rs11082304 | T | G | 0.043033 | 0.00236018 | 3.06E-74 | 0.513659 | -0.0495479 | 0.0333222 | 0.137032 | 0.512397 |
|  | rs11083767 | C | T | 0.0502525 | 0.0024998 | 7.87E-90 | 0.338754 | -0.00307978 | 0.0352471 | 0.930372 | 0.34154 |
|  | rs11086023 | T | C | -0.0293348 | 0.00270282 | 1.94E-27 | 0.280967 | 0.0438594 | 0.0381909 | 0.250793 | 0.281133 |
|  | rs11120218 | A | G | 0.041934 | 0.00366103 | 2.27E-30 | 0.118818 | -0.0668422 | 0.0514564 | 0.193941 | 0.121805 |
|  | rs11121012 | G | A | -0.0261326 | 0.00248379 | 6.96E-26 | 0.645612 | -0.0557445 | 0.0350312 | 0.111546 | 0.644324 |
|  | rs11122449 | T | C | -0.0264677 | 0.00271934 | 2.19E-22 | 0.745285 | -0.0304885 | 0.038348 | 0.426585 | 0.744263 |
|  | rs11123325 | A | G | -0.020984 | 0.00340538 | 7.19E-10 | 0.858629 | -0.0279069 | 0.0481091 | 0.561864 | 0.858971 |
|  | rs111400190 | A | C | 0.017586 | 0.00303423 | 6.80E-09 | 0.195939 | -0.0453151 | 0.0426937 | 0.288508 | 0.197685 |
|  | rs111419866 | T | C | -0.0585292 | 0.00586259 | 1.81E-23 | 0.0462596 | -0.148737 | 0.0823614 | 0.0709316 | 0.0463773 |
|  | rs111425801 | A | G | 0.052973 | 0.00631271 | 4.82E-17 | 0.036453 | -0.0345552 | 0.0888718 | 0.697408 | 0.0364922 |
|  | rs111430408 | T | C | 0.0255483 | 0.00461279 | 3.05E-08 | 0.0713537 | 0.0235639 | 0.0656086 | 0.719477 | 0.0702679 |
|  | rs11143739 | A | G | -0.0201041 | 0.00332493 | 1.48E-09 | 0.179787 | -0.0426502 | 0.0471065 | 0.365255 | 0.179032 |
|  | rs111456533 | A | G | 0.0194427 | 0.00320318 | 1.28E-09 | 0.162454 | 0.0284777 | 0.0452376 | 0.529014 | 0.162254 |
|  | rs11149742 | C | G | -0.0282694 | 0.00248152 | 4.64E-30 | 0.355274 | 0.0228234 | 0.0350876 | 0.51539 | 0.354006 |
|  | rs111631026 | T | A | 0.0368423 | 0.00665871 | 3.15E-08 | 0.0339095 | -0.0840281 | 0.0943295 | 0.37304 | 0.0335275 |
|  | rs111755462 | G | A | 0.0537442 | 0.00867734 | 5.89E-10 | 0.0198513 | 0.0301561 | 0.123707 | 0.807409 | 0.0196539 |
|  | rs111827912 | A | C | 0.0371541 | 0.0055629 | 2.41E-11 | 0.0529651 | 0.116603 | 0.07868 | 0.138342 | 0.0529991 |
|  | rs111941366 | T | C | 0.025444 | 0.00236819 | 6.38E-27 | 0.447576 | -0.0104765 | 0.033407 | 0.753824 | 0.445157 |
|  | rs1121985 | C | A | -0.0198726 | 0.0024564 | 5.98E-16 | 0.625695 | -0.0173506 | 0.0347046 | 0.617109 | 0.623037 |
|  | rs11224303 | A | G | 0.0318693 | 0.00398592 | 1.29E-15 | 0.0974904 | -0.00878789 | 0.0559827 | 0.875264 | 0.0985438 |
|  | rs11235689 | T | C | 0.031624 | 0.00239557 | 8.85E-40 | 0.414095 | 0.0221794 | 0.0338895 | 0.512814 | 0.413021 |
|  | rs112527491 | A | G | 0.0223322 | 0.003833 | 5.67E-09 | 0.128924 | -0.117171 | 0.0540159 | 0.0300677 | 0.129025 |
|  | rs112661098 | T | G | -0.0340841 | 0.00379752 | 2.84E-19 | 0.108999 | -0.101462 | 0.0532193 | 0.0565861 | 0.111636 |
|  | rs112698054 | T | C | 0.0374563 | 0.00650285 | 8.42E-09 | 0.0395692 | -0.103315 | 0.0909795 | 0.256132 | 0.0398492 |
|  | rs113176001 | C | G | -0.0276972 | 0.00256159 | 3.03E-27 | 0.423912 | -0.0949284 | 0.0361655 | 0.00866922 | 0.427588 |
|  | rs113720761 | G | C | 0.0355314 | 0.00605731 | 4.47E-09 | 0.0406879 | 0.0609215 | 0.0860538 | 0.478978 | 0.0396933 |
|  | rs113734692 | G | A | 0.0471777 | 0.00780043 | 1.47E-09 | 0.0233795 | 0.00691331 | 0.109262 | 0.949549 | 0.0237032 |
|  | rs113856656 | A | G | -0.0331336 | 0.004861 | 9.36E-12 | 0.0631874 | -0.0798434 | 0.0689008 | 0.246531 | 0.0621994 |
|  | rs113872186 | T | C | -0.0662163 | 0.0121229 | 4.71E-08 | 0.0103021 | 0.134206 | 0.170036 | 0.429947 | 0.0104316 |
|  | rs114263200 | G | A | -0.0521195 | 0.00810116 | 1.25E-10 | 0.0230385 | -0.146311 | 0.114427 | 0.201023 | 0.0230576 |
|  | rs114694170 | C | T | -0.118791 | 0.00504333 | 1.42E-122 | 0.0594237 | 0.0916384 | 0.0714179 | 0.199447 | 0.0583487 |
|  | rs114971318 | T | C | 0.0577608 | 0.0104138 | 2.92E-08 | 0.0144135 | 0.156066 | 0.147839 | 0.291129 | 0.0143951 |
|  | rs115274585 | G | A | 0.0195937 | 0.00349603 | 2.09E-08 | 0.130993 | 0.0397096 | 0.049178 | 0.419399 | 0.132858 |
|  | rs115340571 | T | C | -0.0287949 | 0.00495752 | 6.32E-09 | 0.0625338 | 0.0246857 | 0.0704938 | 0.726201 | 0.0616997 |
|  | rs115352378 | T | A | 0.0481143 | 0.0065296 | 1.73E-13 | 0.0376454 | -0.0520451 | 0.0919945 | 0.571569 | 0.0377317 |
|  | rs11553699 | G | A | 0.136388 | 0.00359224 | 1.00E-200 | 0.135346 | 0.00764362 | 0.0505984 | 0.879925 | 0.135323 |
|  | rs115871261 | A | T | 0.0456124 | 0.00795534 | 9.84E-09 | 0.0248889 | -0.0866543 | 0.110988 | 0.434948 | 0.0259031 |
|  | rs115978492 | A | C | -0.0400456 | 0.00609664 | 5.09E-11 | 0.0404541 | -0.0252158 | 0.0849616 | 0.766627 | 0.0416597 |
|  | rs11598602 | A | G | -0.0261779 | 0.00305927 | 1.16E-17 | 0.183886 | -0.0389397 | 0.0430961 | 0.366231 | 0.183953 |
|  | rs116167629 | T | C | 0.0268051 | 0.00422956 | 2.34E-10 | 0.0861553 | 0.0141882 | 0.0598608 | 0.812641 | 0.0852367 |
|  | rs116497749 | C | A | 0.0351261 | 0.00633307 | 2.92E-08 | 0.0360345 | 0.138167 | 0.0897147 | 0.123543 | 0.0356795 |
|  | rs116669894 | A | G | 0.0628193 | 0.00890208 | 1.71E-12 | 0.017906 | 0.0982677 | 0.126903 | 0.438722 | 0.0180806 |
|  | rs116778355 | T | A | 0.151384 | 0.00870676 | 1.11E-67 | 0.0211318 | -0.0235295 | 0.121658 | 0.846641 | 0.0213001 |
|  | rs11696739 | A | G | -0.0257305 | 0.00243273 | 3.85E-26 | 0.378153 | 0.0170854 | 0.0343798 | 0.619217 | 0.377915 |
|  | rs117038461 | T | C | 0.0394752 | 0.00721631 | 4.50E-08 | 0.027797 | 0.0267225 | 0.101884 | 0.793102 | 0.0276069 |
|  | rs11703986 | A | G | -0.0224439 | 0.00302088 | 1.09E-13 | 0.189116 | 0.0577312 | 0.0429208 | 0.178604 | 0.187367 |
|  | rs117105320 | A | G | -0.0600084 | 0.009119 | 4.69E-11 | 0.0183078 | -0.127484 | 0.130088 | 0.327097 | 0.0179845 |
|  | rs117106707 | C | T | 0.046317 | 0.00742898 | 4.53E-10 | 0.0269815 | -0.0420032 | 0.104085 | 0.686547 | 0.0273551 |
|  | rs117159862 | A | C | 0.0420663 | 0.00672872 | 4.06E-10 | 0.0334791 | -0.0713637 | 0.0966238 | 0.460167 | 0.0326741 |
|  | rs1171602 | G | T | -0.0153109 | 0.00239991 | 1.78E-10 | 0.584546 | 0.0253698 | 0.0338631 | 0.453744 | 0.583403 |
|  | rs117172295 | A | T | 0.120786 | 0.0107846 | 4.13E-29 | 0.0124905 | -0.0232952 | 0.152636 | 0.878699 | 0.0127958 |
|  | rs11731274 | G | T | -0.064039 | 0.00310046 | 1.01E-94 | 0.176277 | 0.0582954 | 0.0441086 | 0.18629 | 0.175638 |
|  | rs117491068 | A | G | 0.121686 | 0.0138919 | 1.97E-18 | 0.00804125 | 0.331235 | 0.198045 | 0.0944213 | 0.00790264 |
|  | rs117517474 | C | T | 0.0846253 | 0.0118666 | 9.95E-13 | 0.010355 | 0.15786 | 0.169111 | 0.350578 | 0.0101684 |
|  | rs117615061 | G | A | 0.247011 | 0.00904094 | 3.52E-164 | 0.018398 | 0.0818959 | 0.128026 | 0.522381 | 0.0181528 |
|  | rs11767227 | G | C | 0.0250369 | 0.00272432 | 3.94E-20 | 0.25445 | 0.0364673 | 0.0384331 | 0.342696 | 0.254452 |
|  | rs117960804 | C | T | -0.0457481 | 0.00820069 | 2.43E-08 | 0.0211915 | -0.0496997 | 0.116972 | 0.67092 | 0.0208197 |
|  | rs118088320 | A | G | -0.0483413 | 0.0063871 | 3.78E-14 | 0.036471 | -0.124438 | 0.0903203 | 0.168285 | 0.0357096 |
|  | rs118146369 | T | C | -0.0363781 | 0.00484785 | 6.21E-14 | 0.064031 | -0.00075893 | 0.0683891 | 0.991146 | 0.0641494 |
|  | rs118157290 | C | A | -0.0561905 | 0.0094597 | 2.85E-09 | 0.0158998 | 0.0277012 | 0.134358 | 0.836655 | 0.016003 |
|  | rs1182197 | C | A | -0.018353 | 0.00244307 | 5.83E-14 | 0.377931 | 0.0451561 | 0.034541 | 0.191105 | 0.376481 |
|  | rs11841319 | T | C | 0.0231806 | 0.00390619 | 2.95E-09 | 0.102576 | -0.0882319 | 0.0553187 | 0.110718 | 0.101872 |
|  | rs11868244 | C | T | -0.0287449 | 0.00361354 | 1.80E-15 | 0.122447 | -0.00959877 | 0.0508789 | 0.85036 | 0.122915 |
|  | rs11937741 | T | C | -0.0232599 | 0.00396892 | 4.62E-09 | 0.104285 | 0.044221 | 0.0561886 | 0.431276 | 0.104407 |
|  | rs11965156 | G | A | 0.0217864 | 0.00276354 | 3.19E-15 | 0.242437 | 0.0978139 | 0.0391585 | 0.0124934 | 0.240634 |
|  | rs1199955 | G | A | -0.0336999 | 0.00385054 | 2.11E-18 | 0.894879 | 0.0465623 | 0.0548709 | 0.396116 | 0.895997 |
|  | rs12041331 | A | G | -0.0389963 | 0.00426952 | 6.66E-20 | 0.0840996 | -0.0449267 | 0.0597303 | 0.451955 | 0.0858835 |
|  | rs12046329 | C | T | 0.0461203 | 0.00236126 | 6.51E-85 | 0.526381 | -0.10487 | 0.0333143 | 0.00164456 | 0.526029 |
|  | rs12128766 | T | C | -0.0195578 | 0.00236871 | 1.50E-16 | 0.483702 | 0.00198134 | 0.0334565 | 0.952776 | 0.482434 |
|  | rs12169371 | T | C | 0.0407245 | 0.0047296 | 7.30E-18 | 0.0673627 | 0.0938376 | 0.0658773 | 0.154322 | 0.0687465 |
|  | rs12202135 | T | C | -0.0297897 | 0.00360188 | 1.34E-16 | 0.129225 | 0.0889021 | 0.0507671 | 0.0799153 | 0.12979 |
|  | rs12209838 | T | G | 0.0217413 | 0.00321959 | 1.45E-11 | 0.16089 | 0.0194058 | 0.0454458 | 0.669373 | 0.159673 |
|  | rs12210538 | G | A | 0.0194829 | 0.00278482 | 2.64E-12 | 0.236109 | -0.00599355 | 0.0393329 | 0.878887 | 0.236342 |
|  | rs12267227 | C | A | 0.0142562 | 0.00259788 | 4.08E-08 | 0.296226 | 0.00853038 | 0.0367304 | 0.816349 | 0.296398 |
|  | rs12289583 | T | C | 0.0316257 | 0.00564998 | 2.18E-08 | 0.0457894 | 0.0741136 | 0.0798411 | 0.353271 | 0.0455988 |
|  | rs12404622 | C | T | 0.057278 | 0.00302766 | 8.84E-80 | 0.187941 | 0.0356937 | 0.0429044 | 0.405445 | 0.186757 |
|  | rs12405515 | T | G | -0.0315551 | 0.00238918 | 8.12E-40 | 0.567681 | -0.0376673 | 0.0337028 | 0.263724 | 0.568339 |
|  | rs12423880 | T | C | -0.0268274 | 0.00354211 | 3.63E-14 | 0.129734 | 0.0374426 | 0.0498756 | 0.452821 | 0.130594 |
|  | rs12445050 | T | C | 0.0272739 | 0.00343777 | 2.14E-15 | 0.137961 | 0.0335968 | 0.048568 | 0.489096 | 0.137399 |
|  | rs12447809 | T | G | -0.0192673 | 0.00303922 | 2.31E-10 | 0.190836 | 0.0105532 | 0.0429298 | 0.805818 | 0.191982 |
|  | rs12564806 | T | C | 0.0288585 | 0.00278128 | 3.22E-25 | 0.236862 | 0.0379581 | 0.0393384 | 0.334589 | 0.23644 |
|  | rs12578356 | A | G | -0.0141135 | 0.00247676 | 1.21E-08 | 0.358152 | 0.0451846 | 0.035001 | 0.196721 | 0.356346 |
|  | rs12594925 | A | G | 0.0223487 | 0.00354846 | 3.02E-10 | 0.127581 | -0.0715353 | 0.0500614 | 0.153018 | 0.128172 |
|  | rs12637288 | A | G | 0.0150927 | 0.002369 | 1.88E-10 | 0.478641 | 0.00531192 | 0.0333971 | 0.873627 | 0.479754 |
|  | rs12640722 | T | A | 0.0225718 | 0.00258172 | 2.28E-18 | 0.700124 | 0.0206012 | 0.0364163 | 0.571588 | 0.699113 |
|  | rs12649139 | C | G | -0.021694 | 0.00347502 | 4.30E-10 | 0.133387 | 0.0503758 | 0.0491158 | 0.305054 | 0.134124 |
|  | rs12665537 | G | A | -0.0142041 | 0.00251589 | 1.65E-08 | 0.332041 | -0.063122 | 0.0355741 | 0.0760011 | 0.330012 |
|  | rs12666334 | T | G | 0.0953518 | 0.00241565 | 1.00E-200 | 0.420612 | 0.00878679 | 0.0341463 | 0.796926 | 0.418393 |
|  | rs1271272 | A | G | -0.0150649 | 0.00255744 | 3.85E-09 | 0.312282 | -0.0245327 | 0.0360922 | 0.49668 | 0.311936 |
|  | rs12729981 | C | T | -0.0676406 | 0.00699045 | 3.83E-22 | 0.0299289 | -0.0188339 | 0.0986386 | 0.848574 | 0.0298985 |
|  | rs12741781 | G | T | 0.0185369 | 0.00252909 | 2.32E-13 | 0.324681 | 0.046061 | 0.0356056 | 0.195789 | 0.326852 |
|  | rs12909047 | A | G | -0.0230177 | 0.00237995 | 4.01E-22 | 0.50473 | 0.000759194 | 0.0336437 | 0.981997 | 0.500967 |
|  | rs12938273 | A | G | -0.0476636 | 0.00237219 | 9.62E-90 | 0.555886 | -0.055596 | 0.0334675 | 0.0966741 | 0.554529 |
|  | rs12997003 | T | C | -0.0215605 | 0.00243479 | 8.39E-19 | 0.384465 | -0.0181248 | 0.034432 | 0.598614 | 0.382096 |
|  | rs13033725 | G | C | 0.0244579 | 0.00252734 | 3.79E-22 | 0.330957 | -0.0297342 | 0.0356037 | 0.403638 | 0.331382 |
|  | rs13039242 | G | A | -0.0315698 | 0.00356353 | 8.10E-19 | 0.128787 | 0.00106842 | 0.0502702 | 0.983043 | 0.128686 |
|  | rs13099273 | T | A | -0.0145804 | 0.00240208 | 1.28E-09 | 0.506653 | 0.0144749 | 0.0339197 | 0.669568 | 0.506301 |
|  | rs13107325 | T | C | -0.0467969 | 0.00449752 | 2.37E-25 | 0.0747867 | 0.073001 | 0.0634155 | 0.249669 | 0.0749125 |
|  | rs131190 | T | G | -0.026706 | 0.00337388 | 2.47E-15 | 0.857344 | 0.0307675 | 0.0479119 | 0.520764 | 0.858329 |
|  | rs13162910 | C | T | -0.0452435 | 0.00700427 | 1.05E-10 | 0.0305732 | 0.164022 | 0.0994778 | 0.0991813 | 0.03012 |
|  | rs13233747 | A | G | -0.0293558 | 0.00248849 | 4.12E-32 | 0.346604 | -0.029488 | 0.0351821 | 0.401945 | 0.348491 |
|  | rs13279707 | T | C | -0.0316306 | 0.00544997 | 6.49E-09 | 0.0502349 | -0.118944 | 0.077145 | 0.123116 | 0.0496217 |
|  | rs13294558 | A | G | 0.0339154 | 0.00542782 | 4.15E-10 | 0.0498131 | -0.0520902 | 0.0766441 | 0.496734 | 0.0496601 |
|  | rs13320485 | T | C | -0.053241 | 0.00926894 | 9.25E-09 | 0.0164764 | 0.0354073 | 0.128961 | 0.783655 | 0.0168385 |
|  | rs13340461 | T | C | 0.026583 | 0.00265937 | 1.60E-23 | 0.27255 | -0.0248325 | 0.0375175 | 0.50804 | 0.274783 |
|  | rs135016 | C | T | -0.0403477 | 0.00495848 | 4.06E-16 | 0.0655207 | -0.134415 | 0.0700406 | 0.0549718 | 0.0651117 |
|  | rs1372770 | G | A | -0.0166361 | 0.00268748 | 6.01E-10 | 0.265361 | -0.0600343 | 0.0380539 | 0.114654 | 0.264421 |
|  | rs1373218 | G | A | 0.0163928 | 0.002379 | 5.56E-12 | 0.546722 | -0.034718 | 0.0336428 | 0.30209 | 0.547756 |
|  | rs137983047 | A | G | 0.0695094 | 0.0105618 | 4.67E-11 | 0.0147629 | -0.166283 | 0.150412 | 0.268935 | 0.0146084 |
|  | rs138186139 | C | G | -0.0449058 | 0.00811551 | 3.14E-08 | 0.0227928 | 0.0431153 | 0.115193 | 0.70819 | 0.0223058 |
|  | rs1388295 | C | G | -0.0361897 | 0.00661903 | 4.57E-08 | 0.0329851 | -0.0560788 | 0.0933985 | 0.548223 | 0.0333072 |
|  | rs138843544 | C | T | -0.0384435 | 0.00697059 | 3.49E-08 | 0.0338611 | 0.0487522 | 0.0981964 | 0.619558 | 0.0336806 |
|  | rs139012432 | T | A | -0.0293095 | 0.00483514 | 1.35E-09 | 0.0641588 | 0.0773141 | 0.0681019 | 0.256262 | 0.0645257 |
|  | rs139435626 | C | A | -0.0669154 | 0.00896068 | 8.18E-14 | 0.0189001 | 0.142877 | 0.127345 | 0.261878 | 0.0185663 |
|  | rs139700185 | A | G | -0.0416688 | 0.00755254 | 3.45E-08 | 0.030225 | -0.153118 | 0.107364 | 0.153822 | 0.0300513 |
|  | rs139725501 | T | C | 0.030262 | 0.00503681 | 1.88E-09 | 0.066423 | -0.0146437 | 0.0720085 | 0.838853 | 0.065092 |
|  | rs139960758 | A | G | 0.068193 | 0.00853583 | 1.36E-15 | 0.0230236 | 0.161525 | 0.120146 | 0.178815 | 0.0232902 |
|  | rs140002699 | C | T | 0.0617657 | 0.0103707 | 2.59E-09 | 0.015397 | -0.0792097 | 0.146927 | 0.589811 | 0.0152406 |
|  | rs140003256 | T | C | -0.045558 | 0.00787773 | 7.34E-09 | 0.0234248 | -0.0994396 | 0.11244 | 0.376491 | 0.0230791 |
|  | rs140554024 | A | G | 0.0733209 | 0.0128739 | 1.23E-08 | 0.0090538 | -0.105273 | 0.18582 | 0.57103 | 0.00904523 |
|  | rs140799803 | A | G | -0.0200135 | 0.00245171 | 3.28E-16 | 0.393711 | -0.0725351 | 0.0346427 | 0.0362769 | 0.392373 |
|  | rs140999406 | C | T | 0.0580077 | 0.00644489 | 2.26E-19 | 0.0349507 | 0.0198429 | 0.0912043 | 0.827768 | 0.035603 |
|  | rs141133167 | G | A | 0.0737172 | 0.0120997 | 1.11E-09 | 0.0117339 | 0.385379 | 0.170111 | 0.0234844 | 0.0116925 |
|  | rs141469619 | G | A | 0.0848077 | 0.0124528 | 9.75E-12 | 0.010142 | 0.0571628 | 0.176495 | 0.746031 | 0.00993311 |
|  | rs1420476 | A | T | 0.0401628 | 0.0057971 | 4.27E-12 | 0.956837 | 0.0172256 | 0.0811774 | 0.831953 | 0.956139 |
|  | rs142104915 | T | C | -0.0895413 | 0.0154829 | 7.33E-09 | 0.00613758 | 0.16234 | 0.20973 | 0.438906 | 0.00649717 |
|  | rs142279800 | G | A | 0.0597494 | 0.00903142 | 3.70E-11 | 0.019629 | 0.118831 | 0.128583 | 0.355405 | 0.0190818 |
|  | rs1426138 | A | G | 0.016949 | 0.00309338 | 4.28E-08 | 0.177387 | -0.0426122 | 0.0436558 | 0.329018 | 0.176507 |
|  | rs1430039 | A | G | -0.0153294 | 0.00258291 | 2.94E-09 | 0.70361 | -0.0354633 | 0.0365041 | 0.331304 | 0.702769 |
|  | rs143204357 | A | G | -0.05731 | 0.00885199 | 9.54E-11 | 0.0203187 | 0.164262 | 0.124563 | 0.187267 | 0.0205477 |
|  | rs143359317 | G | C | -0.0280607 | 0.00374001 | 6.26E-14 | 0.115481 | 0.0818897 | 0.0529129 | 0.121711 | 0.114697 |
|  | rs1436138 | G | A | 0.0164473 | 0.00247064 | 2.80E-11 | 0.358886 | -0.0147074 | 0.0349011 | 0.67346 | 0.360652 |
|  | rs144232975 | A | G | 0.0520765 | 0.00872541 | 2.40E-09 | 0.0190995 | -0.0637985 | 0.12349 | 0.605415 | 0.0190561 |
|  | rs144409768 | G | A | 0.124311 | 0.00610095 | 3.11E-92 | 0.0411552 | -0.00884855 | 0.0869419 | 0.918935 | 0.0405749 |
|  | rs1448319 | G | A | -0.0628142 | 0.00516382 | 4.89E-34 | 0.944646 | -0.191265 | 0.0716019 | 0.00755718 | 0.942097 |
|  | rs145057384 | T | C | 0.109198 | 0.0157538 | 4.17E-12 | 0.00644528 | 0.327936 | 0.225076 | 0.145115 | 0.00648155 |
|  | rs145171343 | T | C | -0.196318 | 0.00977848 | 1.33E-89 | 0.0160371 | -0.070199 | 0.139533 | 0.614894 | 0.0156804 |
|  | rs146215574 | G | A | -0.0802436 | 0.0104155 | 1.32E-14 | 0.0133723 | 0.0515886 | 0.147808 | 0.727071 | 0.0132009 |
|  | rs147187166 | G | A | -0.073705 | 0.00952213 | 9.94E-15 | 0.0161769 | 0.261699 | 0.135836 | 0.0540319 | 0.0159056 |
|  | rs147994358 | G | C | 0.0656491 | 0.00827093 | 2.07E-15 | 0.0243053 | 0.321617 | 0.116509 | 0.00577231 | 0.0244034 |
|  | rs148221695 | C | T | 0.0365838 | 0.00559573 | 6.25E-11 | 0.0473033 | -0.0945766 | 0.0796354 | 0.234983 | 0.0467251 |
|  | rs148299382 | T | C | 0.0226788 | 0.00376683 | 1.74E-09 | 0.110479 | -0.0426473 | 0.0532809 | 0.423465 | 0.109498 |
|  | rs148377259 | C | T | 0.0468845 | 0.00679271 | 5.13E-12 | 0.0351784 | 0.148163 | 0.0961992 | 0.123519 | 0.0353388 |
|  | rs148561432 | A | G | -0.0544097 | 0.00571946 | 1.86E-21 | 0.0491679 | 0.0138325 | 0.0809333 | 0.864293 | 0.0492782 |
|  | rs150087 | T | C | 0.0190122 | 0.00256494 | 1.24E-13 | 0.698002 | -0.0138321 | 0.0362432 | 0.702724 | 0.699953 |
|  | rs1505307 | C | T | 0.0160461 | 0.00247991 | 9.78E-11 | 0.651515 | 0.0385871 | 0.0349773 | 0.269939 | 0.650087 |
|  | rs1532675 | C | T | 0.0242247 | 0.00394446 | 8.19E-10 | 0.0999652 | 0.0266605 | 0.0560107 | 0.634083 | 0.0994088 |
|  | rs156356 | C | T | 0.0190152 | 0.00243852 | 6.32E-15 | 0.457475 | -0.0137626 | 0.0344593 | 0.689608 | 0.46067 |
|  | rs1644005 | C | T | -0.0181483 | 0.00245817 | 1.55E-13 | 0.361708 | 0.0469707 | 0.0347116 | 0.176001 | 0.360936 |
|  | rs16860216 | A | G | 0.0154652 | 0.00276784 | 2.31E-08 | 0.242941 | 0.0557459 | 0.0391273 | 0.154235 | 0.243597 |
|  | rs16865390 | A | C | 0.0405034 | 0.00718611 | 1.74E-08 | 0.027974 | -0.17298 | 0.0986818 | 0.0796178 | 0.0300116 |
|  | rs16875506 | G | A | 0.0349408 | 0.00259425 | 2.45E-41 | 0.296235 | -0.0425787 | 0.03651 | 0.243525 | 0.297117 |
|  | rs16937003 | A | G | -0.07432 | 0.00858303 | 4.78E-18 | 0.019688 | -0.013105 | 0.119295 | 0.912525 | 0.0206641 |
|  | rs16954792 | C | T | -0.0183458 | 0.00326285 | 1.88E-08 | 0.155064 | 0.0190245 | 0.0461639 | 0.68026 | 0.154272 |
|  | rs17138723 | G | C | 0.0198565 | 0.00287507 | 4.98E-12 | 0.216506 | -0.00879161 | 0.0407369 | 0.829132 | 0.216069 |
|  | rs1719285 | A | T | -0.0854476 | 0.00324461 | 1.07E-152 | 0.15683 | -0.0382814 | 0.0457892 | 0.403135 | 0.159234 |
|  | rs17232863 | C | G | -0.0223117 | 0.00374931 | 2.67E-09 | 0.119159 | 0.0298412 | 0.0525742 | 0.570305 | 0.121369 |
|  | rs17296412 | A | C | -0.0158837 | 0.00275492 | 8.14E-09 | 0.245976 | 0.0913352 | 0.0387671 | 0.0184731 | 0.246001 |
|  | rs17463814 | A | G | -0.0191605 | 0.00285199 | 1.84E-11 | 0.220702 | 0.0798872 | 0.0402248 | 0.0470316 | 0.221659 |
|  | rs17497197 | C | T | -0.0158365 | 0.00276789 | 1.06E-08 | 0.241965 | 0.00782887 | 0.0390755 | 0.841205 | 0.241823 |
|  | rs17572109 | A | G | -0.0453559 | 0.00281511 | 2.22E-58 | 0.233783 | 0.011093 | 0.0397709 | 0.780304 | 0.233449 |
|  | rs17598137 | A | C | -0.0249756 | 0.00386035 | 9.83E-11 | 0.106147 | 0.0373217 | 0.0544239 | 0.492865 | 0.1057 |
|  | rs17664531 | C | T | -0.0365621 | 0.00325536 | 2.89E-29 | 0.156734 | -0.0350134 | 0.0460523 | 0.447078 | 0.15597 |
|  | rs17689615 | T | C | -0.0301142 | 0.00295008 | 1.84E-24 | 0.20511 | 0.0236553 | 0.0416915 | 0.570449 | 0.204901 |
|  | rs17694832 | A | G | 0.0152994 | 0.00240947 | 2.16E-10 | 0.403779 | -0.043995 | 0.0341079 | 0.197094 | 0.399581 |
|  | rs17726337 | C | G | -0.0376032 | 0.00632049 | 2.69E-09 | 0.0373634 | 0.00760725 | 0.0897167 | 0.932427 | 0.0371196 |
|  | rs17825630 | A | G | 0.0202265 | 0.0035026 | 7.72E-09 | 0.143827 | 0.0559767 | 0.0494103 | 0.257259 | 0.143803 |
|  | rs17843783 | A | G | -0.0742948 | 0.00773827 | 7.97E-22 | 0.0239521 | 0.0105484 | 0.110325 | 0.923828 | 0.0239786 |
|  | rs1797077 | T | C | 0.0340761 | 0.00237104 | 8.02E-47 | 0.479595 | 0.0102199 | 0.033469 | 0.760095 | 0.479401 |
|  | rs1801282 | G | C | -0.0263625 | 0.00362269 | 3.42E-13 | 0.120321 | 0.042107 | 0.0514073 | 0.412738 | 0.119575 |
|  | rs1813046 | A | G | 0.0239502 | 0.00267893 | 3.90E-19 | 0.267288 | 0.00382848 | 0.0378887 | 0.919514 | 0.26654 |
|  | rs185645716 | T | C | -0.026266 | 0.00421587 | 4.66E-10 | 0.0863865 | -0.0280973 | 0.0598389 | 0.638678 | 0.0857997 |
|  | rs1870028 | T | C | 0.0333346 | 0.0045232 | 1.71E-13 | 0.0747219 | 0.0188089 | 0.0640397 | 0.768982 | 0.0743489 |
|  | rs187115451 | A | T | 0.0475319 | 0.00603895 | 3.53E-15 | 0.0487714 | 0.0370165 | 0.0853274 | 0.664422 | 0.0487736 |
|  | rs1874309 | T | C | 0.0167119 | 0.00292277 | 1.08E-08 | 0.7957 | 0.00467547 | 0.0412799 | 0.909822 | 0.796777 |
|  | rs188854094 | G | A | -0.0814221 | 0.0103184 | 3.01E-15 | 0.0133986 | -0.0166543 | 0.14723 | 0.909938 | 0.0129754 |
|  | rs189047226 | C | T | 0.0407722 | 0.0061369 | 3.06E-11 | 0.0404909 | 0.134164 | 0.0865686 | 0.12119 | 0.0404689 |
|  | rs189174 | C | G | 0.01897 | 0.00251264 | 4.37E-14 | 0.335971 | 0.0569503 | 0.0354426 | 0.108091 | 0.339115 |
|  | rs1914037 | G | A | 0.0171751 | 0.00269044 | 1.73E-10 | 0.264834 | -0.0339158 | 0.0379339 | 0.37128 | 0.267251 |
|  | rs192565874 | A | G | -0.0246872 | 0.00433338 | 1.22E-08 | 0.0820725 | -0.0769904 | 0.0615242 | 0.210794 | 0.0809992 |
|  | rs1933165 | T | A | -0.0138065 | 0.00236175 | 5.04E-09 | 0.508121 | -0.00629533 | 0.0334213 | 0.850592 | 0.505295 |
|  | rs1961319 | T | G | -0.0181859 | 0.00328304 | 3.04E-08 | 0.152653 | -0.0284993 | 0.0464851 | 0.53982 | 0.152071 |
|  | rs1978504 | C | T | 0.0246683 | 0.00236839 | 2.12E-25 | 0.473803 | -0.00574303 | 0.0335188 | 0.863958 | 0.470815 |
|  | rs1980532 | G | A | -0.0559743 | 0.00238649 | 1.47E-121 | 0.447203 | -0.00537413 | 0.0337164 | 0.87336 | 0.447094 |
|  | rs199568356 | C | T | 0.0288636 | 0.00303882 | 2.15E-21 | 0.22729 | 0.00608823 | 0.0430149 | 0.887445 | 0.224928 |
|  | rs200891 | G | T | -0.0186081 | 0.00245035 | 3.11E-14 | 0.60661 | -0.0261623 | 0.034682 | 0.45064 | 0.607596 |
|  | rs2012011 | A | G | 0.0259671 | 0.00346117 | 6.28E-14 | 0.13481 | -0.0873028 | 0.0488277 | 0.0737802 | 0.135226 |
|  | rs2014850 | A | G | 0.0326802 | 0.00266511 | 1.47E-34 | 0.715951 | 0.0281584 | 0.0375588 | 0.453427 | 0.713766 |
|  | rs2022003 | T | A | -0.0213702 | 0.00263506 | 5.08E-16 | 0.277901 | -0.0107486 | 0.0371386 | 0.772261 | 0.279054 |
|  | rs2048084 | T | G | 0.0250898 | 0.00242653 | 4.69E-25 | 0.405352 | 0.00762365 | 0.034246 | 0.823835 | 0.405302 |
|  | rs2048894 | A | G | -0.0173912 | 0.00272364 | 1.71E-10 | 0.252972 | 0.00502491 | 0.038437 | 0.895988 | 0.255043 |
|  | rs207419 | G | A | -0.0216094 | 0.00241904 | 4.16E-19 | 0.595076 | 0.0664435 | 0.0341405 | 0.0516333 | 0.595255 |
|  | rs2097598 | G | C | 0.0264222 | 0.00252157 | 1.09E-25 | 0.377431 | 0.00856249 | 0.0356183 | 0.810023 | 0.37626 |
|  | rs2099001 | G | A | -0.0460609 | 0.00313614 | 8.06E-49 | 0.172798 | 0.000996321 | 0.0441999 | 0.982016 | 0.172918 |
|  | rs210152 | A | G | -0.0196309 | 0.00298305 | 4.68E-11 | 0.805754 | 0.0341423 | 0.0423308 | 0.41992 | 0.808726 |
|  | rs2111174 | T | G | 0.0137664 | 0.00239155 | 8.61E-09 | 0.579398 | 0.00734781 | 0.0337001 | 0.827402 | 0.579208 |
|  | rs214065 | A | G | 0.0416699 | 0.00238477 | 2.44E-68 | 0.427757 | 0.0235892 | 0.033739 | 0.484448 | 0.425762 |
|  | rs2173356 | A | G | 0.0144687 | 0.00242015 | 2.26E-09 | 0.602848 | 0.0215818 | 0.0342018 | 0.52803 | 0.601476 |
|  | rs2176455 | C | T | -0.014608 | 0.00244919 | 2.46E-09 | 0.620729 | -0.0861987 | 0.0345426 | 0.0125806 | 0.621265 |
|  | rs2230585 | A | G | 0.0198404 | 0.00242066 | 2.49E-16 | 0.391378 | -0.00489476 | 0.034149 | 0.886025 | 0.391478 |
|  | rs2235895 | G | C | 0.0182777 | 0.0025441 | 6.77E-13 | 0.675973 | 0.00779646 | 0.0359613 | 0.828363 | 0.676391 |
|  | rs2236142 | C | G | -0.0142146 | 0.00253735 | 2.12E-08 | 0.680127 | 0.0401704 | 0.0357781 | 0.261538 | 0.676634 |
|  | rs224049 | A | T | -0.0169098 | 0.00240905 | 2.23E-12 | 0.600105 | -0.0498576 | 0.0339968 | 0.142501 | 0.599222 |
|  | rs2288367 | T | G | -0.028628 | 0.00383665 | 8.56E-14 | 0.105782 | -0.0965857 | 0.0544292 | 0.0759766 | 0.105404 |
|  | rs2291813 | T | G | -0.0455925 | 0.00630619 | 4.85E-13 | 0.0363797 | -0.0414635 | 0.089381 | 0.642722 | 0.0361969 |
|  | rs2293176 | A | G | 0.0176343 | 0.00250337 | 1.87E-12 | 0.334636 | 0.0104406 | 0.0353317 | 0.767609 | 0.334328 |
|  | rs229576 | T | A | -0.0221093 | 0.00314492 | 2.07E-12 | 0.175661 | -0.0126389 | 0.0442254 | 0.775043 | 0.176429 |
|  | rs2326842 | T | C | -0.0231671 | 0.00298816 | 9.00E-15 | 0.195255 | 0.00406219 | 0.0421202 | 0.923169 | 0.194727 |
|  | rs2327531 | G | A | -0.0218091 | 0.00290406 | 5.93E-14 | 0.209316 | 0.0651141 | 0.0410851 | 0.112999 | 0.208146 |
|  | rs2392492 | A | G | -0.0315161 | 0.00501759 | 3.36E-10 | 0.0622941 | -0.0375536 | 0.0707544 | 0.595585 | 0.0618307 |
|  | rs2405861 | A | G | -0.0140833 | 0.0023692 | 2.78E-09 | 0.526855 | 0.0259763 | 0.0334869 | 0.437916 | 0.528154 |
|  | rs2424698 | T | C | -0.0202079 | 0.00238236 | 2.22E-17 | 0.437854 | 0.0312236 | 0.0336891 | 0.354022 | 0.435067 |
|  | rs2429511 | C | T | -0.0176727 | 0.00236882 | 8.64E-14 | 0.478926 | -0.00509169 | 0.033448 | 0.879008 | 0.478609 |
|  | rs243332 | T | C | -0.0209197 | 0.00263527 | 2.05E-15 | 0.276533 | -0.0242806 | 0.0371757 | 0.513672 | 0.277882 |
|  | rs2462506 | A | G | 0.0234374 | 0.00264242 | 7.37E-19 | 0.284943 | -0.017226 | 0.03707 | 0.642155 | 0.285658 |
|  | rs2468896 | T | G | 0.016538 | 0.00292263 | 1.53E-08 | 0.786313 | 0.0236456 | 0.0411884 | 0.565911 | 0.784225 |
|  | rs254415 | T | G | 0.0320304 | 0.00257024 | 1.22E-35 | 0.303684 | -0.0340706 | 0.0362913 | 0.347828 | 0.303906 |
|  | rs2562396 | A | C | -0.0211592 | 0.00237516 | 5.20E-19 | 0.459724 | 0.00912017 | 0.0335249 | 0.78559 | 0.459991 |
|  | rs2622637 | T | G | 0.0132005 | 0.00239457 | 3.54E-08 | 0.415414 | 0.0181335 | 0.0338382 | 0.592036 | 0.414162 |
|  | rs2632372 | C | T | -0.0132278 | 0.00235981 | 2.08E-08 | 0.50348 | -0.0378428 | 0.0333463 | 0.256442 | 0.504294 |
|  | rs263978 | C | T | 0.0172361 | 0.00237184 | 3.68E-13 | 0.550254 | 0.0334395 | 0.0335066 | 0.31828 | 0.550795 |
|  | rs268 | G | A | 0.0805915 | 0.00877737 | 4.26E-20 | 0.0184481 | 0.12424 | 0.123112 | 0.312897 | 0.0182189 |
|  | rs2734331 | G | A | 0.0750395 | 0.00691868 | 2.11E-27 | 0.0299912 | -0.0212418 | 0.0956492 | 0.824252 | 0.0312587 |
|  | rs2749110 | C | T | -0.0149384 | 0.00244577 | 1.01E-09 | 0.369227 | 0.0240642 | 0.0345012 | 0.485496 | 0.370389 |
|  | rs2759390 | C | T | 0.0153897 | 0.00249281 | 6.68E-10 | 0.339865 | 0.0219771 | 0.0351601 | 0.531933 | 0.339333 |
|  | rs2761843 | C | T | -0.0194303 | 0.00325277 | 2.33E-09 | 0.157412 | -0.00736058 | 0.045736 | 0.872144 | 0.15772 |
|  | rs2767627 | A | C | -0.0155281 | 0.00237877 | 6.68E-11 | 0.45273 | 0.0123348 | 0.0335897 | 0.713456 | 0.453724 |
|  | rs2789422 | A | G | 0.0235419 | 0.00239462 | 8.32E-23 | 0.416698 | 0.0302457 | 0.0338021 | 0.370901 | 0.419404 |
|  | rs28369829 | A | C | -0.022922 | 0.00408641 | 2.03E-08 | 0.0919259 | -0.0262067 | 0.0576533 | 0.649428 | 0.0919842 |
|  | rs28483039 | A | G | -0.0207781 | 0.00275189 | 4.35E-14 | 0.243005 | 0.0381204 | 0.0387723 | 0.325515 | 0.244013 |
|  | rs28607509 | G | A | 0.0323087 | 0.00402393 | 9.85E-16 | 0.0955243 | 0.0594909 | 0.0572296 | 0.298566 | 0.0942984 |
|  | rs28678897 | A | G | -0.0465746 | 0.00439188 | 2.86E-26 | 0.0787539 | -0.0670937 | 0.0621516 | 0.280357 | 0.0777184 |
|  | rs28806851 | A | G | -0.0135879 | 0.0024354 | 2.42E-08 | 0.387582 | 0.0718624 | 0.0344759 | 0.037121 | 0.385676 |
|  | rs2934691 | A | C | -0.0142486 | 0.00238055 | 2.16E-09 | 0.562829 | -0.012089 | 0.0336087 | 0.719073 | 0.562361 |
|  | rs2950387 | C | T | -0.0658237 | 0.00245128 | 1.14E-158 | 0.366652 | -0.00754997 | 0.0346106 | 0.82732 | 0.366807 |
|  | rs2965180 | C | T | 0.0192771 | 0.0024811 | 7.89E-15 | 0.650344 | 0.01498 | 0.034895 | 0.667713 | 0.650009 |
|  | rs2977491 | A | G | 0.0159123 | 0.00266053 | 2.22E-09 | 0.720139 | 0.015707 | 0.0375872 | 0.676033 | 0.720881 |
|  | rs2979244 | A | T | -0.0310623 | 0.00246055 | 1.58E-36 | 0.628318 | 0.0226763 | 0.0347707 | 0.514293 | 0.627396 |
|  | rs2991216 | G | A | -0.0159881 | 0.00270785 | 3.54E-09 | 0.261565 | -0.0375818 | 0.0383306 | 0.326857 | 0.259942 |
|  | rs3103780 | G | A | -0.0147403 | 0.00268186 | 3.88E-08 | 0.265874 | 0.0344734 | 0.0379141 | 0.363218 | 0.265164 |
|  | rs312824 | T | C | 0.0143753 | 0.00259216 | 2.93E-08 | 0.702143 | 0.0343 | 0.0366478 | 0.349306 | 0.700516 |
|  | rs3132558 | G | C | 0.0163582 | 0.00273704 | 2.28E-09 | 0.74387 | 0.014884 | 0.0386416 | 0.700103 | 0.744608 |
|  | rs3204 | G | A | 0.0154745 | 0.00238474 | 8.65E-11 | 0.46036 | -0.0623121 | 0.0336399 | 0.0639779 | 0.46152 |
|  | rs3212931 | T | G | 0.0532579 | 0.00743746 | 8.04E-13 | 0.0259219 | 0.00913752 | 0.104068 | 0.930033 | 0.0262871 |
|  | rs332035 | C | T | -0.0232964 | 0.00340557 | 7.90E-12 | 0.13958 | -0.116694 | 0.0480992 | 0.0152619 | 0.141108 |
|  | rs333288 | A | G | 0.0276821 | 0.00252706 | 6.40E-28 | 0.324661 | -0.0476656 | 0.0356734 | 0.181494 | 0.326216 |
|  | rs34038797 | G | C | 0.030803 | 0.00238948 | 5.15E-38 | 0.480052 | -0.0289869 | 0.0337351 | 0.390202 | 0.479165 |
|  | rs34060709 | A | G | 0.0398578 | 0.00542978 | 2.13E-13 | 0.0588992 | 0.0194636 | 0.0766354 | 0.799514 | 0.058679 |
|  | rs34137317 | T | C | 0.0534409 | 0.00877487 | 1.13E-09 | 0.0184081 | 0.126312 | 0.125734 | 0.31509 | 0.0180234 |
|  | rs34161672 | A | G | -0.0143994 | 0.00252632 | 1.20E-08 | 0.334078 | 0.0208993 | 0.0356337 | 0.557536 | 0.332873 |
|  | rs34188294 | A | G | -0.0172233 | 0.0029181 | 3.59E-09 | 0.208343 | 0.0135758 | 0.0411846 | 0.741679 | 0.20812 |
|  | rs34228166 | G | A | -0.0243494 | 0.00412008 | 3.43E-09 | 0.0905398 | 0.0276994 | 0.0583438 | 0.634957 | 0.090642 |
|  | rs342304 | G | C | 0.0413334 | 0.00443645 | 1.21E-20 | 0.922078 | -0.0535805 | 0.0634596 | 0.398489 | 0.923383 |
|  | rs34297269 | A | G | -0.033965 | 0.00466238 | 3.23E-13 | 0.0739484 | -0.0659433 | 0.0658818 | 0.316859 | 0.0730957 |
|  | rs34363565 | G | A | -0.0244764 | 0.00442603 | 3.20E-08 | 0.0802886 | -0.0601526 | 0.0627624 | 0.337852 | 0.0808265 |
|  | rs34410760 | G | A | 0.0415808 | 0.00689362 | 1.62E-09 | 0.0372937 | -0.0860864 | 0.0976063 | 0.37779 | 0.0368084 |
|  | rs34480360 | A | G | 0.0165069 | 0.00269042 | 8.50E-10 | 0.26214 | -0.0466563 | 0.0379111 | 0.218444 | 0.264054 |
|  | rs34592828 | A | G | 0.0546791 | 0.00565625 | 4.19E-22 | 0.0455916 | 0.0469484 | 0.0804709 | 0.559609 | 0.0448256 |
|  | rs34633608 | T | A | -0.0267938 | 0.0038785 | 4.91E-12 | 0.103591 | -0.0280389 | 0.0544846 | 0.606819 | 0.104662 |
|  | rs346745 | C | T | 0.0135981 | 0.00242934 | 2.18E-08 | 0.588088 | 0.074181 | 0.0342527 | 0.0303347 | 0.586082 |
|  | rs34773782 | C | A | 0.0172536 | 0.00298932 | 7.85E-09 | 0.195033 | -0.00521241 | 0.0420383 | 0.901322 | 0.196303 |
|  | rs34834842 | G | A | 0.102657 | 0.00618111 | 6.42E-62 | 0.0380204 | -0.0455429 | 0.0885874 | 0.607181 | 0.0372154 |
|  | rs35188965 | T | C | -0.0289362 | 0.00239417 | 1.27E-33 | 0.58164 | -0.00383592 | 0.0337584 | 0.909532 | 0.579729 |
|  | rs35548455 | T | C | -0.0266571 | 0.00469234 | 1.34E-08 | 0.0678375 | 0.0606352 | 0.0666208 | 0.362741 | 0.0675247 |
|  | rs35564315 | A | C | -0.02562 | 0.00318638 | 8.98E-16 | 0.165601 | -0.0356431 | 0.0449867 | 0.428185 | 0.165175 |
|  | rs3557 | G | T | -0.0558826 | 0.00415522 | 3.20E-41 | 0.0904563 | -0.0591899 | 0.0588427 | 0.314463 | 0.0898737 |
|  | rs35669853 | A | G | -0.0268331 | 0.00298431 | 2.45E-19 | 0.194971 | 0.0440098 | 0.042295 | 0.298087 | 0.192982 |
|  | rs3742003 | A | G | -0.0310426 | 0.0029102 | 1.47E-26 | 0.20983 | 0.0640875 | 0.0411275 | 0.119171 | 0.21059 |
|  | rs3748136 | A | G | 0.0868722 | 0.00289078 | 3.73E-198 | 0.211353 | 0.117866 | 0.0409667 | 0.00401328 | 0.208669 |
|  | rs3761260 | A | G | 0.157692 | 0.00342912 | 1.00E-200 | 0.137604 | -0.0970889 | 0.0486139 | 0.04581 | 0.137813 |
|  | rs3767812 | A | G | -0.0350365 | 0.00284664 | 8.34E-35 | 0.221191 | -0.039939 | 0.0400499 | 0.318652 | 0.223706 |
|  | rs3770098 | A | C | -0.016354 | 0.00238948 | 7.70E-12 | 0.424925 | -0.0239613 | 0.0337626 | 0.47789 | 0.423707 |
|  | rs3791936 | G | T | -0.0154972 | 0.00246025 | 3.00E-10 | 0.374497 | 0.0557795 | 0.0346911 | 0.10786 | 0.376584 |
|  | rs3800227 | G | A | -0.0199669 | 0.0027158 | 1.96E-13 | 0.744372 | -0.0493643 | 0.0383572 | 0.198107 | 0.743005 |
|  | rs3804749 | T | C | 0.0396884 | 0.00241647 | 1.35E-60 | 0.593088 | -0.0523526 | 0.034061 | 0.124287 | 0.590945 |
|  | rs3810353 | G | C | 0.0349885 | 0.00412076 | 2.06E-17 | 0.0902737 | -0.0340287 | 0.0581012 | 0.558091 | 0.091464 |
|  | rs3816257 | C | A | 0.0174336 | 0.00242155 | 6.06E-13 | 0.390646 | 0.0365037 | 0.0342295 | 0.286225 | 0.389441 |
|  | rs3828933 | C | T | -0.0141026 | 0.0023846 | 3.34E-09 | 0.438227 | 0.0456059 | 0.0336977 | 0.175932 | 0.440145 |
|  | rs3845498 | C | T | -0.0139337 | 0.00240931 | 7.33E-09 | 0.59708 | 0.0306943 | 0.034045 | 0.36728 | 0.592772 |
|  | rs3896096 | A | G | 0.0135311 | 0.00238813 | 1.46E-08 | 0.448791 | -0.0102455 | 0.033722 | 0.761263 | 0.448687 |
|  | rs3937015 | C | T | -0.0904019 | 0.00869093 | 2.45E-25 | 0.981004 | -0.0557667 | 0.121946 | 0.647451 | 0.980513 |
|  | rs40270 | C | A | 0.0204655 | 0.00291897 | 2.37E-12 | 0.772845 | 0.072015 | 0.039765 | 0.0701375 | 0.771886 |
|  | rs4099245 | A | G | -0.0173885 | 0.00240325 | 4.65E-13 | 0.414117 | 0.00636505 | 0.0338981 | 0.851057 | 0.414836 |
|  | rs41284294 | C | T | 0.0631186 | 0.0112845 | 2.23E-08 | 0.0110523 | 0.019278 | 0.159161 | 0.903594 | 0.0110881 |
|  | rs41316548 | T | C | 0.0361583 | 0.00490562 | 1.70E-13 | 0.0621122 | -0.0156844 | 0.0699901 | 0.822684 | 0.0608098 |
|  | rs415895 | G | C | 0.0310392 | 0.00246962 | 3.21E-36 | 0.645205 | 0.0459685 | 0.0348363 | 0.186984 | 0.646872 |
|  | rs4236051 | T | C | -0.102176 | 0.0126128 | 5.47E-16 | 0.00887397 | 0.0059542 | 0.176048 | 0.973019 | 0.00915599 |
|  | rs4267257 | G | A | 0.0228189 | 0.00304075 | 6.19E-14 | 0.185816 | 0.00385332 | 0.0428166 | 0.92829 | 0.187602 |
|  | rs441233 | A | G | -0.0210784 | 0.00262236 | 9.16E-16 | 0.290728 | 0.0491949 | 0.0370197 | 0.183886 | 0.291454 |
|  | rs4425772 | T | C | -0.0370377 | 0.00267754 | 1.66E-43 | 0.264316 | 0.00548095 | 0.0378086 | 0.884738 | 0.26544 |
|  | rs4529894 | G | C | -0.028889 | 0.00286881 | 7.55E-24 | 0.217266 | -0.019043 | 0.0405894 | 0.638955 | 0.216636 |
|  | rs4549095 | G | A | 0.0157477 | 0.00258237 | 1.07E-09 | 0.697988 | -0.0747393 | 0.0365047 | 0.04062 | 0.699921 |
|  | rs45563932 | T | C | 0.0163473 | 0.00283987 | 8.60E-09 | 0.222202 | 0.0967556 | 0.0401808 | 0.0160398 | 0.220646 |
|  | rs4584571 | T | A | 0.0191634 | 0.00260958 | 2.09E-13 | 0.290316 | 0.059893 | 0.0368423 | 0.104022 | 0.290985 |
|  | rs4671117 | A | G | 0.0161043 | 0.00243868 | 4.02E-11 | 0.621607 | 0.0264979 | 0.0343714 | 0.440749 | 0.618436 |
|  | rs4711890 | G | C | -0.0410847 | 0.00267502 | 3.23E-53 | 0.267057 | -0.0366047 | 0.0377753 | 0.33254 | 0.267638 |
|  | rs4721764 | A | G | -0.0225652 | 0.00287743 | 4.44E-15 | 0.21404 | -0.0410674 | 0.0408747 | 0.315034 | 0.212378 |
|  | rs4744227 | T | C | 0.0156323 | 0.00236967 | 4.21E-11 | 0.460798 | 0.0477468 | 0.0334743 | 0.153762 | 0.456866 |
|  | rs4751648 | T | C | -0.0159602 | 0.0023714 | 1.70E-11 | 0.547843 | 0.00710214 | 0.033482 | 0.832015 | 0.547349 |
|  | rs4760335 | C | T | -0.0211035 | 0.00252674 | 6.73E-17 | 0.326408 | -0.00119757 | 0.0356839 | 0.973228 | 0.323785 |
|  | rs4783186 | C | T | 0.0570868 | 0.0035811 | 3.44E-57 | 0.876218 | 0.030956 | 0.0506307 | 0.54093 | 0.876173 |
|  | rs4804833 | G | A | -0.0135008 | 0.00242534 | 2.60E-08 | 0.618818 | -0.0141495 | 0.0342613 | 0.679614 | 0.615763 |
|  | rs4808319 | A | G | -0.0169703 | 0.00237119 | 8.27E-13 | 0.517057 | -0.0271249 | 0.0334929 | 0.418014 | 0.51845 |
|  | rs4814776 | A | C | -0.0194463 | 0.00252197 | 1.25E-14 | 0.328291 | 0.0487656 | 0.0355273 | 0.16987 | 0.329857 |
|  | rs4880075 | C | G | -0.0190178 | 0.00302341 | 3.18E-10 | 0.812467 | -0.0212367 | 0.0427347 | 0.61923 | 0.812622 |
|  | rs4900573 | T | C | -0.0265314 | 0.00315975 | 4.61E-17 | 0.170271 | -0.0317413 | 0.0444417 | 0.475089 | 0.171158 |
|  | rs4911243 | A | G | 0.0138694 | 0.00253412 | 4.43E-08 | 0.323107 | 0.0156515 | 0.0357309 | 0.661357 | 0.323379 |
|  | rs4925684 | T | C | -0.0360945 | 0.00310877 | 3.69E-31 | 0.178324 | 0.0174281 | 0.0438056 | 0.690739 | 0.178742 |
|  | rs4952073 | C | T | -0.115982 | 0.00260733 | 1.00E-200 | 0.702925 | 0.011315 | 0.0369411 | 0.759378 | 0.702841 |
|  | rs4958244 | G | A | -0.0409067 | 0.00239436 | 2.05E-65 | 0.580195 | -0.0487715 | 0.0338653 | 0.149822 | 0.581082 |
|  | rs4966015 | T | G | 0.029593 | 0.00336602 | 1.48E-18 | 0.143519 | 0.0773531 | 0.0474629 | 0.103152 | 0.143567 |
|  | rs507666 | A | G | -0.0284168 | 0.00305357 | 1.33E-20 | 0.18413 | -0.0182562 | 0.0428351 | 0.669963 | 0.184758 |
|  | rs5112 | G | C | 0.0173869 | 0.00253443 | 6.88E-12 | 0.5321 | 0.0452703 | 0.0358744 | 0.20698 | 0.533552 |
|  | rs540909 | C | T | 0.0520447 | 0.00273697 | 1.39E-80 | 0.754334 | -0.0160187 | 0.0385926 | 0.678089 | 0.753786 |
|  | rs55710545 | C | G | -0.0325578 | 0.00308806 | 5.51E-26 | 0.178557 | -0.010202 | 0.0437451 | 0.815594 | 0.178733 |
|  | rs55770470 | T | C | 0.0410007 | 0.0054993 | 8.97E-14 | 0.0513636 | -0.0874892 | 0.0782401 | 0.263476 | 0.0505617 |
|  | rs55903042 | A | G | 0.0172731 | 0.00269507 | 1.47E-10 | 0.261941 | 0.0463789 | 0.037928 | 0.2214 | 0.264125 |
|  | rs55913685 | G | A | -0.0194517 | 0.00244208 | 1.65E-15 | 0.3772 | -0.0561587 | 0.0344457 | 0.103027 | 0.377423 |
|  | rs55953372 | T | C | -0.0183286 | 0.00302331 | 1.34E-09 | 0.191426 | -0.0364447 | 0.0427137 | 0.393531 | 0.191698 |
|  | rs55966522 | A | G | -0.0380593 | 0.00536276 | 1.28E-12 | 0.0533618 | 0.0527516 | 0.0761104 | 0.488251 | 0.0532377 |
|  | rs55970551 | T | C | -0.0632578 | 0.00823858 | 1.62E-14 | 0.0211519 | 0.047412 | 0.116659 | 0.684438 | 0.0212617 |
|  | rs56043070 | A | G | 0.13669 | 0.00457175 | 3.61E-196 | 0.0715168 | 0.00957377 | 0.0643236 | 0.881682 | 0.071697 |
|  | rs56094005 | G | A | 0.0440053 | 0.0057757 | 2.56E-14 | 0.0438154 | -0.163943 | 0.0813292 | 0.0438218 | 0.043374 |
|  | rs56231935 | C | T | -0.0193631 | 0.00238226 | 4.38E-16 | 0.563798 | -0.00222854 | 0.0337096 | 0.94729 | 0.563576 |
|  | rs56353063 | C | G | 0.0156242 | 0.00273931 | 1.17E-08 | 0.251479 | 0.0143626 | 0.0388177 | 0.711381 | 0.249375 |
|  | rs564273193 | T | C | -0.0623385 | 0.00977268 | 1.79E-10 | 0.0165804 | 0.222528 | 0.138499 | 0.108116 | 0.0162414 |
|  | rs56956502 | A | G | -0.0340138 | 0.00273799 | 2.00E-35 | 0.247746 | -0.043207 | 0.0386511 | 0.263621 | 0.24778 |
|  | rs574339 | C | T | 0.0145074 | 0.00263931 | 3.87E-08 | 0.277007 | 0.0178899 | 0.0371924 | 0.63051 | 0.279134 |
|  | rs57556994 | C | T | -0.0475787 | 0.00448324 | 2.63E-26 | 0.0752379 | 0.116979 | 0.0633129 | 0.0646547 | 0.0754265 |
|  | rs5758910 | C | G | -0.0273077 | 0.00252986 | 3.70E-27 | 0.676098 | 0.0175228 | 0.0358072 | 0.624583 | 0.67673 |
|  | rs5758968 | C | T | 0.0892786 | 0.00235925 | 1.00E-200 | 0.491971 | 0.0274015 | 0.0333668 | 0.411521 | 0.490433 |
|  | rs57843631 | T | C | 0.225532 | 0.00890894 | 2.91E-141 | 0.0192005 | 0.0527372 | 0.124276 | 0.671305 | 0.0198011 |
|  | rs580840 | G | C | -0.0171125 | 0.00277893 | 7.38E-10 | 0.758215 | -0.0271439 | 0.0392107 | 0.488776 | 0.758371 |
|  | rs58245033 | T | C | 0.0204738 | 0.00340528 | 1.83E-09 | 0.142166 | -0.0323024 | 0.048154 | 0.502338 | 0.142099 |
|  | rs58323965 | C | A | -0.0233066 | 0.00355289 | 5.39E-11 | 0.126442 | 0.037806 | 0.0498558 | 0.448268 | 0.128189 |
|  | rs58455864 | C | T | 0.0222261 | 0.00297741 | 8.35E-14 | 0.197176 | 0.0722301 | 0.042092 | 0.0861609 | 0.195787 |
|  | rs58672755 | A | G | 0.0734199 | 0.0102418 | 7.59E-13 | 0.0156147 | -0.0544952 | 0.14299 | 0.703121 | 0.0158136 |
|  | rs59228823 | C | G | -0.0190979 | 0.00276129 | 4.64E-12 | 0.245248 | 0.0816957 | 0.0388459 | 0.0354593 | 0.244845 |
|  | rs59242558 | C | T | -0.0168215 | 0.00264271 | 1.95E-10 | 0.298268 | -0.026202 | 0.0373179 | 0.482599 | 0.29768 |
|  | rs59882870 | A | G | -0.0185046 | 0.00320149 | 7.48E-09 | 0.164444 | -0.0407388 | 0.0452015 | 0.367445 | 0.163848 |
|  | rs6003007 | A | G | 0.0347581 | 0.00417091 | 7.88E-17 | 0.0878121 | 0.0806095 | 0.0589582 | 0.171552 | 0.0885118 |
|  | rs6026450 | G | A | -0.0256351 | 0.00244534 | 1.04E-25 | 0.631472 | -0.0378681 | 0.0345716 | 0.273361 | 0.63243 |
|  | rs6026791 | C | T | 0.284336 | 0.00866203 | 1.00E-200 | 0.01917 | -0.0525239 | 0.119798 | 0.661069 | 0.0200425 |
|  | rs6026837 | T | G | 0.0260865 | 0.00264622 | 6.37E-23 | 0.273536 | -0.0020099 | 0.037397 | 0.957138 | 0.272872 |
|  | rs6052122 | A | G | -0.0183374 | 0.00252227 | 3.60E-13 | 0.416062 | 0.0871653 | 0.0356087 | 0.0143704 | 0.417944 |
|  | rs60609026 | C | A | 0.0167777 | 0.00293329 | 1.07E-08 | 0.207047 | 0.00684884 | 0.0414786 | 0.868852 | 0.206147 |
|  | rs606552 | G | A | 0.0604271 | 0.00275109 | 7.40E-107 | 0.246965 | -0.0376944 | 0.0387561 | 0.330749 | 0.249453 |
|  | rs6070629 | T | G | 0.0332968 | 0.00279925 | 1.28E-32 | 0.763174 | -0.0579351 | 0.0395263 | 0.142721 | 0.761778 |
|  | rs6084506 | T | C | -0.0211972 | 0.00248349 | 1.40E-17 | 0.380039 | -0.0632445 | 0.0350753 | 0.0713707 | 0.379063 |
|  | rs6086540 | C | G | 0.03613 | 0.0023669 | 1.37E-52 | 0.506741 | 0.0371243 | 0.0334122 | 0.266525 | 0.507545 |
|  | rs61237993 | A | G | -0.0198839 | 0.00354889 | 2.11E-08 | 0.12699 | -0.00704086 | 0.0500002 | 0.888015 | 0.127818 |
|  | rs6124259 | A | G | 0.0170991 | 0.00244613 | 2.75E-12 | 0.618715 | -0.0293315 | 0.034571 | 0.396191 | 0.61838 |
|  | rs61269268 | C | A | 0.0302009 | 0.00313231 | 5.36E-22 | 0.17399 | -0.0419719 | 0.0442767 | 0.343157 | 0.173432 |
|  | rs61730474 | A | G | -0.0541366 | 0.00863946 | 3.70E-10 | 0.0190031 | -0.133897 | 0.123145 | 0.276899 | 0.0185396 |
|  | rs61748681 | A | G | -0.0784152 | 0.0111203 | 1.77E-12 | 0.012199 | 0.299864 | 0.157223 | 0.0564885 | 0.0120147 |
|  | rs61750929 | T | C | 0.0515774 | 0.00518916 | 2.82E-23 | 0.0551214 | -0.014927 | 0.0725523 | 0.836993 | 0.0558818 |
|  | rs61751937 | C | G | 0.0818035 | 0.00700606 | 1.71E-31 | 0.0292065 | 0.0910398 | 0.0995039 | 0.360225 | 0.0293491 |
|  | rs61851769 | T | C | 0.0278392 | 0.00282082 | 5.70E-23 | 0.236631 | 0.0221185 | 0.0398875 | 0.579223 | 0.235227 |
|  | rs619874 | C | T | 0.0155625 | 0.00244429 | 1.93E-10 | 0.615087 | -0.0156821 | 0.0344999 | 0.649429 | 0.613522 |
|  | rs62022924 | G | A | 0.0158716 | 0.0028053 | 1.54E-08 | 0.237483 | 0.0218883 | 0.0392728 | 0.577295 | 0.235286 |
|  | rs62059826 | T | C | -0.0290405 | 0.00309943 | 7.32E-21 | 0.176567 | -0.0804633 | 0.0437921 | 0.0661515 | 0.175595 |
|  | rs62067167 | T | C | -0.020459 | 0.00313504 | 6.77E-11 | 0.176543 | 0.00678708 | 0.0441461 | 0.877814 | 0.17813 |
|  | rs62109598 | T | C | -0.0256167 | 0.00284279 | 2.05E-19 | 0.223159 | 0.0022575 | 0.0401451 | 0.955156 | 0.222124 |
|  | rs62116961 | C | G | 0.0146589 | 0.00244181 | 1.94E-09 | 0.377308 | -0.000883053 | 0.034546 | 0.979607 | 0.375568 |
|  | rs62162753 | C | A | 0.0150751 | 0.00238541 | 2.62E-10 | 0.430093 | 0.0212971 | 0.0336994 | 0.527406 | 0.427652 |
|  | rs62191340 | A | G | 0.03533 | 0.00236998 | 3.06E-50 | 0.539151 | -0.06343 | 0.0334989 | 0.0582921 | 0.540743 |
|  | rs62231637 | A | C | -0.0775125 | 0.00867647 | 4.14E-19 | 0.0197665 | -0.0308617 | 0.123022 | 0.801919 | 0.0195551 |
|  | rs62246446 | G | C | 0.0485592 | 0.003097 | 2.18E-55 | 0.175793 | -0.0165013 | 0.0438689 | 0.706806 | 0.173734 |
|  | rs62267575 | A | G | 0.0286924 | 0.00381418 | 5.39E-14 | 0.108839 | 0.0598648 | 0.0543683 | 0.270854 | 0.107287 |
|  | rs62291089 | G | C | 0.021523 | 0.00314463 | 7.69E-12 | 0.194589 | -0.0737434 | 0.0446526 | 0.098637 | 0.193363 |
|  | rs62379580 | C | T | 0.0344545 | 0.00250734 | 5.88E-43 | 0.665533 | -0.057223 | 0.0353707 | 0.105704 | 0.664305 |
|  | rs62387962 | T | G | 0.0309978 | 0.00338157 | 4.90E-20 | 0.14355 | -0.0667303 | 0.0477956 | 0.162666 | 0.143062 |
|  | rs62493228 | A | C | 0.0454402 | 0.00594631 | 2.15E-14 | 0.041169 | 0.0927938 | 0.0845776 | 0.272579 | 0.0406576 |
|  | rs62543170 | G | A | 0.0243484 | 0.00238183 | 1.58E-24 | 0.436185 | -0.00164995 | 0.0336357 | 0.960877 | 0.434724 |
|  | rs631106 | A | C | -0.0482905 | 0.00247483 | 9.51E-85 | 0.354716 | 0.0428558 | 0.0349559 | 0.220201 | 0.352699 |
|  | rs639836 | A | G | 0.0187676 | 0.00250719 | 7.14E-14 | 0.666201 | -0.0193831 | 0.0354421 | 0.584451 | 0.666806 |
|  | rs640527 | A | G | 0.033916 | 0.00435547 | 6.88E-15 | 0.919912 | 0.120202 | 0.061367 | 0.0501418 | 0.919721 |
|  | rs6443617 | G | A | 0.0193846 | 0.00274143 | 1.54E-12 | 0.749529 | 0.0689215 | 0.0388024 | 0.0756972 | 0.750911 |
|  | rs6492945 | C | T | 0.0148314 | 0.00237623 | 4.34E-10 | 0.458365 | 0.0644292 | 0.0335196 | 0.0545884 | 0.455937 |
|  | rs650169 | T | C | 0.0494568 | 0.00881221 | 2.00E-08 | 0.0181856 | 0.0893478 | 0.120321 | 0.457736 | 0.0194199 |
|  | rs651166 | A | G | -0.0520837 | 0.003485 | 1.74E-50 | 0.136108 | -0.0555523 | 0.0488547 | 0.255499 | 0.138455 |
|  | rs6547599 | T | A | -0.0139961 | 0.00244829 | 1.09E-08 | 0.602967 | -0.0274675 | 0.0346063 | 0.427362 | 0.602987 |
|  | rs6556405 | C | T | 0.0310435 | 0.00273442 | 7.26E-30 | 0.248806 | -0.0193418 | 0.0386159 | 0.616459 | 0.251647 |
|  | rs6558407 | T | C | -0.048461 | 0.00238896 | 1.95E-91 | 0.424477 | -0.0515734 | 0.033754 | 0.126533 | 0.42414 |
|  | rs6602909 | C | T | 0.0168545 | 0.00252488 | 2.47E-11 | 0.325236 | 0.0437138 | 0.0356719 | 0.220409 | 0.327689 |
|  | rs66530629 | A | G | 0.0406674 | 0.00273189 | 4.20E-50 | 0.2493 | 0.00163262 | 0.0384588 | 0.966139 | 0.252032 |
|  | rs666404 | G | A | 0.0213743 | 0.00236806 | 1.79E-19 | 0.52985 | 0.0199265 | 0.0334595 | 0.551482 | 0.529918 |
|  | rs6671349 | C | T | 0.0237306 | 0.00247345 | 8.51E-22 | 0.349612 | 0.0647778 | 0.0349344 | 0.0637001 | 0.349217 |
|  | rs667805 | G | A | -0.023086 | 0.00345302 | 2.30E-11 | 0.134566 | 0.0114311 | 0.0487927 | 0.814768 | 0.135064 |
|  | rs66793829 | C | G | 0.0526062 | 0.00317732 | 1.51E-61 | 0.167873 | -0.00379311 | 0.0447967 | 0.932521 | 0.168829 |
|  | rs6681849 | T | G | -0.0299417 | 0.00240756 | 1.68E-35 | 0.421662 | -0.0464713 | 0.0339532 | 0.171098 | 0.42115 |
|  | rs668674 | G | A | -0.0137075 | 0.00250637 | 4.53E-08 | 0.337631 | -0.0157765 | 0.0353614 | 0.65549 | 0.336885 |
|  | rs6698181 | T | C | -0.0138132 | 0.00242041 | 1.15E-08 | 0.389187 | 0.0371806 | 0.0342468 | 0.277627 | 0.386276 |
|  | rs6703318 | A | G | 0.122154 | 0.0077642 | 9.40E-56 | 0.0242606 | 0.207068 | 0.109103 | 0.0577085 | 0.0247301 |
|  | rs670863 | T | A | 0.0297504 | 0.00522022 | 1.21E-08 | 0.942956 | 0.046525 | 0.0738481 | 0.528689 | 0.943779 |
|  | rs6709953 | A | G | 0.0338201 | 0.00489906 | 5.09E-12 | 0.0619311 | -0.085828 | 0.0687254 | 0.211718 | 0.0637109 |
|  | rs671339 | G | A | -0.0389801 | 0.00268297 | 8.23E-48 | 0.736515 | 0.0106334 | 0.0378841 | 0.778953 | 0.735903 |
|  | rs6784200 | G | A | -0.0159335 | 0.00244751 | 7.52E-11 | 0.629826 | -0.00699872 | 0.0345552 | 0.839497 | 0.6301 |
|  | rs6796069 | T | C | 0.0138592 | 0.00251978 | 3.80E-08 | 0.325953 | 0.0224398 | 0.0356526 | 0.529087 | 0.326233 |
|  | rs6796837 | T | C | -0.0203246 | 0.00256512 | 2.32E-15 | 0.304205 | -0.064935 | 0.0361089 | 0.072129 | 0.306318 |
|  | rs6808932 | C | A | 0.0133197 | 0.00242799 | 4.12E-08 | 0.386384 | 0.0392788 | 0.0342041 | 0.250818 | 0.387639 |
|  | rs686408 | A | G | -0.0184568 | 0.00276478 | 2.46E-11 | 0.758291 | 0.00803511 | 0.0390368 | 0.83692 | 0.758607 |
|  | rs688181 | C | T | 0.0376038 | 0.00262737 | 1.89E-46 | 0.719258 | 0.0388693 | 0.0371309 | 0.295183 | 0.719266 |
|  | rs6905288 | A | G | 0.0182438 | 0.00238479 | 2.01E-14 | 0.567348 | -0.0337656 | 0.0336974 | 0.316332 | 0.568996 |
|  | rs6906721 | G | A | -0.0459269 | 0.0036649 | 5.11E-36 | 0.119373 | -0.0168604 | 0.0512643 | 0.742238 | 0.121595 |
|  | rs691243 | T | C | -0.0151226 | 0.00255513 | 3.25E-09 | 0.691835 | -0.0412569 | 0.0360797 | 0.252834 | 0.691806 |
|  | rs6912933 | A | G | -0.0162778 | 0.00238529 | 8.86E-12 | 0.5275 | 0.030312 | 0.0336359 | 0.367493 | 0.528412 |
|  | rs6914448 | T | C | -0.016727 | 0.00279817 | 2.26E-09 | 0.764416 | 0.0317306 | 0.0396146 | 0.423142 | 0.764052 |
|  | rs6947609 | C | G | -0.0299646 | 0.00368892 | 4.57E-16 | 0.870796 | 0.00646344 | 0.0520878 | 0.901246 | 0.870999 |
|  | rs6956591 | C | G | 0.0145993 | 0.00238437 | 9.20E-10 | 0.44881 | -0.0703945 | 0.0336862 | 0.0366438 | 0.45103 |
|  | rs6969773 | C | T | 0.0140642 | 0.00237644 | 3.26E-09 | 0.46515 | -0.0645036 | 0.0336025 | 0.0549073 | 0.467395 |
|  | rs6974282 | T | C | -0.0739691 | 0.00299384 | 1.17E-134 | 0.195874 | 0.0572969 | 0.0422257 | 0.174807 | 0.195987 |
|  | rs6993770 | T | A | 0.058895 | 0.00261119 | 1.45E-112 | 0.286014 | -0.0559309 | 0.036899 | 0.129573 | 0.28737 |
|  | rs6994859 | A | G | -0.0177165 | 0.00247614 | 8.39E-13 | 0.36282 | -0.0212315 | 0.0349206 | 0.543192 | 0.362785 |
|  | rs6999008 | A | G | 0.0154781 | 0.00238141 | 8.07E-11 | 0.443825 | 0.0546524 | 0.0336334 | 0.104175 | 0.443574 |
|  | rs7002892 | T | A | -0.0178585 | 0.00236259 | 4.07E-14 | 0.497646 | 0.0335855 | 0.0333623 | 0.314084 | 0.499795 |
|  | rs7024673 | T | C | -0.0169917 | 0.00255378 | 2.86E-11 | 0.314579 | 0.013537 | 0.0361668 | 0.708185 | 0.312234 |
|  | rs7107356 | G | A | 0.0179629 | 0.00236103 | 2.79E-14 | 0.505693 | 0.00291224 | 0.0333478 | 0.93041 | 0.506732 |
|  | rs7114102 | T | A | 0.0265416 | 0.00449926 | 3.66E-09 | 0.922753 | -0.0595877 | 0.0633218 | 0.34669 | 0.922623 |
|  | rs7127443 | G | A | 0.016847 | 0.00281156 | 2.07E-09 | 0.771556 | -9.60E-05 | 0.03978 | 0.998075 | 0.773276 |
|  | rs71531541 | T | C | -0.0156227 | 0.00265985 | 4.27E-09 | 0.728453 | 0.0324488 | 0.0375186 | 0.38711 | 0.726463 |
|  | rs71567501 | A | G | -0.0539435 | 0.00797619 | 1.35E-11 | 0.0245589 | 0.126094 | 0.112657 | 0.263024 | 0.0243121 |
|  | rs7158644 | G | A | 0.0187585 | 0.00316176 | 2.98E-09 | 0.832355 | -0.00266006 | 0.044656 | 0.9525 | 0.831431 |
|  | rs7170637 | A | G | 0.0284306 | 0.00321497 | 9.35E-19 | 0.160141 | 0.049107 | 0.0452915 | 0.278258 | 0.16057 |
|  | rs718444 | T | C | -0.0191173 | 0.00244553 | 5.41E-15 | 0.371058 | -0.0352492 | 0.0345228 | 0.307235 | 0.370914 |
|  | rs718515 | A | G | 0.0165699 | 0.00237677 | 3.14E-12 | 0.552477 | -0.0203871 | 0.0335602 | 0.543533 | 0.552403 |
|  | rs7210383 | A | G | 0.0433888 | 0.00616463 | 1.95E-12 | 0.0385387 | -0.0377925 | 0.0859189 | 0.660037 | 0.0391955 |
|  | rs72665221 | C | A | 0.0281096 | 0.00351182 | 1.21E-15 | 0.129472 | 0.0563585 | 0.0498382 | 0.258127 | 0.127641 |
|  | rs727088 | A | G | 0.0525936 | 0.00236445 | 1.56E-109 | 0.525074 | 0.0327813 | 0.0333759 | 0.326009 | 0.52461 |
|  | rs72710406 | C | A | 0.0293948 | 0.0035179 | 6.52E-17 | 0.130233 | 0.0783098 | 0.0495559 | 0.114054 | 0.130206 |
|  | rs72713739 | C | A | 0.138512 | 0.00626866 | 4.09E-108 | 0.0371684 | 0.0186497 | 0.0885575 | 0.833204 | 0.0371268 |
|  | rs72716236 | G | A | 0.0590275 | 0.0107559 | 4.07E-08 | 0.0134867 | 0.162615 | 0.153153 | 0.288333 | 0.0132791 |
|  | rs7273251 | T | C | 0.137112 | 0.00664457 | 1.51E-94 | 0.0340867 | -0.0689325 | 0.0946348 | 0.466365 | 0.0335228 |
|  | rs72771512 | A | G | -0.0485688 | 0.00756679 | 1.38E-10 | 0.0248951 | -0.128559 | 0.108287 | 0.235145 | 0.0242997 |
|  | rs72772025 | T | C | -0.0149512 | 0.00271214 | 3.54E-08 | 0.257981 | 0.0251699 | 0.0382819 | 0.510867 | 0.258516 |
|  | rs72775795 | A | G | 0.0417877 | 0.00734673 | 1.29E-08 | 0.0272822 | -0.146739 | 0.103408 | 0.155892 | 0.0270477 |
|  | rs72914576 | G | C | 0.0218598 | 0.00306234 | 9.47E-13 | 0.181806 | 0.017787 | 0.0431251 | 0.68001 | 0.183191 |
|  | rs72966959 | T | C | -0.0352484 | 0.00571955 | 7.16E-10 | 0.0446443 | 0.072241 | 0.0814873 | 0.375332 | 0.0440447 |
|  | rs7304273 | C | A | 0.0733744 | 0.0120766 | 1.24E-09 | 0.0098186 | 0.0367951 | 0.174105 | 0.832623 | 0.00979876 |
|  | rs73068668 | A | G | -0.0304562 | 0.00450752 | 1.41E-11 | 0.0757586 | -0.043309 | 0.0634275 | 0.494726 | 0.0756168 |
|  | rs73110139 | G | A | 0.0390601 | 0.00579474 | 1.58E-11 | 0.0438987 | -0.0405554 | 0.0810242 | 0.6167 | 0.0446583 |
|  | rs73205708 | A | G | -0.0189745 | 0.00273906 | 4.29E-12 | 0.248196 | -0.0118539 | 0.038767 | 0.759777 | 0.247032 |
|  | rs733324 | A | G | -0.0411585 | 0.00271557 | 7.13E-52 | 0.258088 | -0.00795753 | 0.0383341 | 0.835554 | 0.257991 |
|  | rs73618656 | A | G | 0.114384 | 0.00470218 | 1.35E-130 | 0.069869 | -0.0676966 | 0.0661885 | 0.30641 | 0.0700203 |
|  | rs73934253 | G | A | -0.0250693 | 0.00378067 | 3.34E-11 | 0.109342 | 0.0739404 | 0.0533444 | 0.165718 | 0.110393 |
|  | rs74432647 | T | C | -0.025825 | 0.00441915 | 5.10E-09 | 0.0806667 | -0.0648336 | 0.0630764 | 0.304017 | 0.079716 |
|  | rs7456677 | T | C | -0.032009 | 0.00574407 | 2.51E-08 | 0.953006 | 0.0233141 | 0.0814404 | 0.77467 | 0.953492 |
|  | rs74846303 | G | A | -0.0459872 | 0.00644011 | 9.30E-13 | 0.0351824 | -0.157685 | 0.0907256 | 0.0822034 | 0.035569 |
|  | rs7503168 | G | A | -0.103908 | 0.00311658 | 1.00E-200 | 0.172459 | -0.0955532 | 0.0439228 | 0.0295944 | 0.173748 |
|  | rs75083064 | C | G | -0.0395934 | 0.00650849 | 1.18E-09 | 0.0381122 | -0.0786959 | 0.0915383 | 0.389952 | 0.0379464 |
|  | rs7537213 | T | G | 0.0225879 | 0.00237016 | 1.58E-21 | 0.540818 | -0.00624644 | 0.0334382 | 0.851813 | 0.540275 |
|  | rs75375644 | A | C | -0.0465818 | 0.00685776 | 1.10E-11 | 0.0315872 | -0.0350654 | 0.0976084 | 0.719411 | 0.0312977 |
|  | rs7538966 | C | T | 0.0133445 | 0.00243907 | 4.47E-08 | 0.624171 | -0.0553824 | 0.0343939 | 0.107346 | 0.622997 |
|  | rs754388 | C | G | -0.0257925 | 0.00302286 | 1.44E-17 | 0.810605 | 0.000193087 | 0.042702 | 0.996392 | 0.810705 |
|  | rs75612655 | T | C | 0.197069 | 0.0128208 | 2.66E-53 | 0.00869506 | -0.0160589 | 0.181833 | 0.929625 | 0.00863749 |
|  | rs75623217 | A | G | 0.074399 | 0.0131114 | 1.39E-08 | 0.0101677 | 0.157252 | 0.181046 | 0.385081 | 0.0104375 |
|  | rs7570428 | C | G | -0.0173488 | 0.00239899 | 4.78E-13 | 0.566128 | 0.0429954 | 0.0338534 | 0.204069 | 0.566637 |
|  | rs7574538 | T | C | 0.0194969 | 0.00281524 | 4.35E-12 | 0.229553 | -0.00299359 | 0.0396514 | 0.939819 | 0.229561 |
|  | rs7574937 | T | C | 0.0692288 | 0.0039887 | 1.89E-67 | 0.0971194 | 0.109846 | 0.0561229 | 0.0503199 | 0.0987522 |
|  | rs76020175 | A | G | 0.0391993 | 0.00689883 | 1.33E-08 | 0.0304833 | 0.0636818 | 0.0971513 | 0.512151 | 0.0305733 |
|  | rs760402 | A | G | 0.0232016 | 0.00255567 | 1.11E-19 | 0.687657 | 0.0256121 | 0.0362183 | 0.479467 | 0.689294 |
|  | rs76257386 | A | G | -0.220012 | 0.00835815 | 1.47E-152 | 0.0206074 | -0.147342 | 0.118616 | 0.21417 | 0.020396 |
|  | rs76378167 | A | G | 0.0445152 | 0.00498069 | 4.00E-19 | 0.0598354 | -0.0190447 | 0.0699779 | 0.785503 | 0.0604977 |
|  | rs7639048 | G | A | -0.0209442 | 0.00237281 | 1.08E-18 | 0.489433 | 0.0109252 | 0.0333505 | 0.743224 | 0.490922 |
|  | rs7640885 | C | A | -0.0231172 | 0.00252769 | 5.96E-20 | 0.668207 | 0.032417 | 0.0357372 | 0.364357 | 0.668807 |
|  | rs76452111 | T | A | 0.133102 | 0.00589583 | 9.06E-113 | 0.043375 | 0.0773179 | 0.0834321 | 0.354074 | 0.0433612 |
|  | rs76565743 | C | T | -0.0424689 | 0.00631175 | 1.72E-11 | 0.0406229 | -0.12246 | 0.0897703 | 0.172523 | 0.040135 |
|  | rs76582570 | G | C | 0.0219446 | 0.0034232 | 1.45E-10 | 0.138696 | 0.0193358 | 0.0481628 | 0.688075 | 0.139589 |
|  | rs76591819 | C | G | 0.032722 | 0.00392053 | 7.07E-17 | 0.100942 | -0.0587885 | 0.0553211 | 0.287928 | 0.100718 |
|  | rs76610863 | A | G | 0.0329965 | 0.00479934 | 6.20E-12 | 0.0646774 | 0.0372887 | 0.0678998 | 0.582887 | 0.0641127 |
|  | rs76794875 | T | C | 0.0477412 | 0.00731674 | 6.81E-11 | 0.0267156 | 0.236978 | 0.102908 | 0.0212897 | 0.0270064 |
|  | rs76900683 | C | G | -0.0420102 | 0.00548279 | 1.83E-14 | 0.0486746 | 0.0405378 | 0.0777719 | 0.6022 | 0.0480346 |
|  | rs7692976 | G | A | 0.0208791 | 0.00242935 | 8.39E-18 | 0.404273 | 0.0142499 | 0.0342471 | 0.677342 | 0.405741 |
|  | rs7696969 | G | T | -0.0366901 | 0.00243303 | 2.28E-51 | 0.619071 | -0.000746639 | 0.0343542 | 0.982661 | 0.618161 |
|  | rs7705526 | A | C | 0.0157659 | 0.00255223 | 6.53E-10 | 0.325355 | -0.0154233 | 0.0359888 | 0.668245 | 0.326588 |
|  | rs7706078 | T | C | 0.0294892 | 0.00238802 | 5.03E-35 | 0.492526 | -0.0668551 | 0.0336865 | 0.0471857 | 0.491617 |
|  | rs77077845 | T | C | 0.0603576 | 0.00910087 | 3.32E-11 | 0.0173156 | -0.0536834 | 0.127909 | 0.674705 | 0.0175478 |
|  | rs77100396 | A | C | 0.0737548 | 0.00959047 | 1.47E-14 | 0.0154007 | -0.153821 | 0.134993 | 0.254507 | 0.0157973 |
|  | rs7732063 | C | G | -0.0214955 | 0.00347374 | 6.10E-10 | 0.138616 | -0.0243751 | 0.0489771 | 0.618707 | 0.139569 |
|  | rs77334232 | T | C | -0.0303763 | 0.00476209 | 1.79E-10 | 0.0667697 | -0.0142114 | 0.0674217 | 0.833055 | 0.0663454 |
|  | rs7754915 | A | G | -0.0152218 | 0.00247513 | 7.76E-10 | 0.648003 | 0.0416206 | 0.0349907 | 0.234252 | 0.651243 |
|  | rs77632732 | G | A | 0.0936569 | 0.0123963 | 4.19E-14 | 0.00948027 | -0.0385149 | 0.173085 | 0.823909 | 0.00985109 |
|  | rs77684563 | C | T | -0.0450402 | 0.0066491 | 1.26E-11 | 0.0327068 | -0.121199 | 0.0928996 | 0.192022 | 0.0328928 |
|  | rs7777102 | G | A | -0.0238057 | 0.00348531 | 8.49E-12 | 0.134431 | 0.0302993 | 0.0495514 | 0.540886 | 0.133316 |
|  | rs77972827 | C | A | 0.0197462 | 0.00349321 | 1.58E-08 | 0.134844 | 0.0118372 | 0.0492783 | 0.810167 | 0.134799 |
|  | rs78000359 | C | A | -0.041933 | 0.00767607 | 4.69E-08 | 0.024226 | 0.0203273 | 0.108996 | 0.852056 | 0.0239951 |
|  | rs7808005 | T | C | 0.0319537 | 0.00264108 | 1.09E-33 | 0.279094 | 0.00455464 | 0.0372858 | 0.902776 | 0.278552 |
|  | rs780803 | A | T | -0.013986 | 0.00243333 | 9.06E-09 | 0.615468 | -0.0121105 | 0.0343652 | 0.724534 | 0.615844 |
|  | rs78229412 | C | T | -0.0258602 | 0.00446111 | 6.76E-09 | 0.0761255 | 0.0621474 | 0.0630906 | 0.3246 | 0.076664 |
|  | rs78278054 | C | G | -0.0358063 | 0.00508638 | 1.93E-12 | 0.0582459 | 0.0493613 | 0.071947 | 0.492663 | 0.0579829 |
|  | rs78648341 | A | G | 0.0706997 | 0.0100778 | 2.30E-12 | 0.0149469 | 0.099439 | 0.142475 | 0.485216 | 0.0148399 |
|  | rs78710257 | A | G | 0.0257721 | 0.0031525 | 2.96E-16 | 0.171038 | 0.0505609 | 0.0446254 | 0.257211 | 0.170031 |
|  | rs78909033 | A | G | -0.0467284 | 0.00344996 | 8.73E-42 | 0.135365 | 0.0151752 | 0.0489437 | 0.756519 | 0.133444 |
|  | rs78947501 | C | G | -0.0469536 | 0.00673251 | 3.08E-12 | 0.0320566 | 0.0786861 | 0.0940753 | 0.40292 | 0.0327621 |
|  | rs7896518 | G | A | -0.0300633 | 0.00241322 | 1.29E-35 | 0.427162 | 0.02788 | 0.0340833 | 0.41336 | 0.427883 |
|  | rs7939541 | T | C | 0.0142834 | 0.00240479 | 2.86E-09 | 0.585787 | -0.0218585 | 0.033907 | 0.519148 | 0.586388 |
|  | rs7949566 | A | G | 0.0225938 | 0.0023902 | 3.32E-21 | 0.421985 | -0.0372648 | 0.0337285 | 0.269227 | 0.422711 |
|  | rs79544064 | A | C | 0.0451417 | 0.00813959 | 2.93E-08 | 0.0215682 | -0.0823272 | 0.115159 | 0.474671 | 0.0214321 |
|  | rs79557869 | C | A | -0.0570384 | 0.00648255 | 1.39E-18 | 0.0364426 | -0.160969 | 0.0918754 | 0.0797682 | 0.0361302 |
|  | rs7958679 | T | C | 0.157259 | 0.00454191 | 1.00E-200 | 0.0737825 | -0.0809918 | 0.0642974 | 0.207798 | 0.0737134 |
|  | rs796063 | T | C | -0.0147525 | 0.00237199 | 4.99E-10 | 0.468379 | 0.0619956 | 0.033485 | 0.0641062 | 0.466936 |
|  | rs7962350 | G | A | -0.0293994 | 0.00490575 | 2.06E-09 | 0.0618805 | -0.00352912 | 0.069045 | 0.959235 | 0.0623395 |
|  | rs79716587 | A | G | 0.0451927 | 0.00357772 | 1.44E-36 | 0.126526 | -0.0128901 | 0.0508675 | 0.799956 | 0.125744 |
|  | rs8017319 | G | A | -0.0314344 | 0.00552034 | 1.24E-08 | 0.0480683 | 0.0280776 | 0.0782803 | 0.719835 | 0.048337 |
|  | rs8028182 | T | G | 0.0248013 | 0.00299124 | 1.12E-16 | 0.193038 | 0.0118055 | 0.0420623 | 0.778967 | 0.196431 |
|  | rs8060638 | T | C | 0.0175239 | 0.00275042 | 1.88E-10 | 0.248222 | 0.00732161 | 0.0388957 | 0.850691 | 0.249064 |
|  | rs8070670 | T | G | 0.0233674 | 0.00309221 | 4.14E-14 | 0.177931 | 0.0681866 | 0.043709 | 0.118756 | 0.176997 |
|  | rs8077177 | T | C | -0.0532905 | 0.0061899 | 7.38E-18 | 0.0389696 | 0.0252596 | 0.0863242 | 0.769818 | 0.0404035 |
|  | rs8098454 | T | C | -0.0274804 | 0.00419423 | 5.69E-11 | 0.0869855 | 0.0482866 | 0.0593578 | 0.415941 | 0.0870089 |
|  | rs8100926 | A | G | 0.0182321 | 0.00240941 | 3.83E-14 | 0.409192 | -0.00288681 | 0.0340723 | 0.932479 | 0.407833 |
|  | rs8106212 | T | C | 0.147577 | 0.0109783 | 3.48E-41 | 0.0120052 | -0.0109363 | 0.153254 | 0.943111 | 0.0121156 |
|  | rs8107247 | T | A | 0.0299441 | 0.00285543 | 1.00E-25 | 0.780026 | 0.0689514 | 0.0403348 | 0.0873635 | 0.779553 |
|  | rs8107977 | T | G | -0.0143536 | 0.00249278 | 8.51E-09 | 0.341302 | 0.0372458 | 0.0351839 | 0.28978 | 0.343059 |
|  | rs813497 | A | G | -0.0193275 | 0.00317176 | 1.10E-09 | 0.16606 | 0.0280458 | 0.0448734 | 0.531972 | 0.165676 |
|  | rs835259 | G | T | -0.013934 | 0.00236883 | 4.05E-09 | 0.475348 | 0.0494536 | 0.0334543 | 0.139342 | 0.477867 |
|  | rs850736 | C | G | 0.034632 | 0.00244691 | 1.83E-45 | 0.373931 | 0.068245 | 0.0345447 | 0.0482048 | 0.374724 |
|  | rs855791 | G | A | 0.0200123 | 0.00238933 | 5.51E-17 | 0.561409 | 0.0046884 | 0.0337415 | 0.889489 | 0.561211 |
|  | rs883394 | T | C | 0.0177942 | 0.00236432 | 5.24E-14 | 0.484658 | -0.00291262 | 0.0333423 | 0.930389 | 0.481144 |
|  | rs883950 | C | G | -0.0148079 | 0.00251719 | 4.04E-09 | 0.672659 | -0.00771625 | 0.0356494 | 0.828638 | 0.674848 |
|  | rs887826 | A | C | -0.0226329 | 0.00339868 | 2.75E-11 | 0.859284 | -0.0420582 | 0.0479247 | 0.380167 | 0.858205 |
|  | rs8887 | C | T | 0.0133983 | 0.00241063 | 2.73E-08 | 0.573498 | 0.0188427 | 0.0340255 | 0.579728 | 0.571259 |
|  | rs892090 | G | T | 0.0807395 | 0.00317521 | 1.66E-142 | 0.834543 | -0.0206172 | 0.04483 | 0.645589 | 0.834311 |
|  | rs896852 | T | G | -0.0189403 | 0.00236345 | 1.12E-15 | 0.511492 | 0.00430869 | 0.0334061 | 0.897374 | 0.512108 |
|  | rs9356100 | T | C | 0.032948 | 0.00508351 | 9.10E-11 | 0.0576668 | -0.0187822 | 0.071679 | 0.793296 | 0.0577647 |
|  | rs9356996 | A | C | 0.0147495 | 0.00239203 | 7.01E-10 | 0.581286 | 0.0390265 | 0.0337927 | 0.24814 | 0.582347 |
|  | rs9357155 | A | G | -0.0256013 | 0.00362604 | 1.66E-12 | 0.120394 | 0.0117787 | 0.0513592 | 0.818605 | 0.119733 |
|  | rs9376091 | T | C | 0.0152746 | 0.0027017 | 1.57E-08 | 0.260613 | -0.0168653 | 0.0381314 | 0.658277 | 0.259713 |
|  | rs9480924 | C | T | -0.0137882 | 0.00238222 | 7.13E-09 | 0.459191 | -0.0171217 | 0.0336859 | 0.61126 | 0.456642 |
|  | rs9534458 | A | G | 0.060612 | 0.0024763 | 3.37E-132 | 0.651889 | 0.03542 | 0.0350096 | 0.311671 | 0.653539 |
|  | rs9616368 | G | C | -0.0179948 | 0.00298285 | 1.61E-09 | 0.195034 | -0.0596241 | 0.0420156 | 0.155872 | 0.196778 |
|  | rs9636612 | G | A | 0.0260393 | 0.00245081 | 2.31E-26 | 0.373181 | -0.00301739 | 0.0346289 | 0.930564 | 0.373747 |
|  | rs964184 | C | G | -0.0811876 | 0.00348092 | 3.16E-120 | 0.867716 | -0.0471729 | 0.048981 | 0.335505 | 0.86637 |
|  | rs9804646 | T | C | -0.0237565 | 0.00429875 | 3.27E-08 | 0.0830294 | -0.0374453 | 0.0607656 | 0.537746 | 0.0834382 |
|  | rs9829114 | A | G | 0.0142221 | 0.00240549 | 3.38E-09 | 0.418377 | 0.0608957 | 0.0340179 | 0.0734362 | 0.416125 |
|  | rs9900637 | A | C | 0.0152248 | 0.00236309 | 1.17E-10 | 0.49813 | 0.0377916 | 0.0333669 | 0.25738 | 0.499152 |
|  | rs9908765 | A | G | -0.0237753 | 0.00236918 | 1.07E-23 | 0.49131 | -0.0307237 | 0.0334443 | 0.358278 | 0.492482 |
|  | rs9914927 | T | C | 0.0212863 | 0.00294155 | 4.62E-13 | 0.202698 | -0.0219233 | 0.0412726 | 0.595292 | 0.204302 |
|  | rs9920 | C | T | -0.0229424 | 0.00384615 | 2.45E-09 | 0.105521 | 0.0580638 | 0.0543012 | 0.284939 | 0.105094 |
|  | rs9925477 | G | T | 0.0294979 | 0.0024052 | 1.43E-34 | 0.467987 | -0.0552012 | 0.0339856 | 0.104322 | 0.466889 |
|  | rs9926137 | C | G | 0.0377779 | 0.00301745 | 5.92E-36 | 0.188721 | -0.0533952 | 0.0424685 | 0.208648 | 0.190415 |
|  | rs997680 | A | G | 0.021687 | 0.00266807 | 4.37E-16 | 0.267362 | -0.0228876 | 0.037749 | 0.54431 | 0.265484 |
|  | rs9997383 | C | A | -0.0258228 | 0.00376873 | 7.30E-12 | 0.113045 | -0.0133992 | 0.0530816 | 0.800712 | 0.113922 |

eaf: effect allele frequency; SE: standard error. SNP, number of single nucleotide polymorphism;

**Table S3. The detail UVMR results of BCIs on Risk of 28-day mortality in sepsis ^a,b^.**

| Exposure | Methods | N SNP | OR (95%CI) | b | SE | P value |
| --- | --- | --- | --- | --- | --- | --- |
| PDW_1_ | IVW | 570 | 1.12(1.01-1.26) | 0.12 | 0.06 | 0.04 |
|  | WM | 570 | 1.09(0.90-1.31) | 0.08 | 0.10 | 0.40 |
|  | MR Egger | 570 | 1.06(0.88-1.28) | 0.06 | 0.10 | 0.52 |
|  | Simple mode | 570 | 1.04(0.65-1.66) | 0.04 | 0.24 | 0.86 |
|  | Weighted mode | 570 | 1.04(0.83-1.31) | 0.04 | 0.12 | 0.72 |
| PDW_2_ | IVW | 99 | 1.23(1.01-1.49) | 0.21 | 0.10 | 0.04 |
|  | WM | 99 | 1.26(0.96-1.67) | 0.23 | 0.14 | 0.10 |
|  | MR Egger | 99 | 1.15(0.80-1.65) | 0.14 | 0.19 | 0.46 |
|  | Simple mode | 99 | 1.62(0.96-2.71) | 0.48 | 0.26 | 0.07 |
|  | Weighted mode | 99 | 1.27(0.92-1.75) | 0.24 | 0.16 | 0.14 |
| PLC | IVW | 719 | 0.97(0.87-1.08) | -0.03 | 0.06 | 0.59 |
|  | WM | 719 | 0.95(0.78-1.15) | -0.05 | 0.10 | 0.60 |
|  | MR Egger | 719 | 0.97(0.78-1.20) | -0.03 | 0.11 | 0.78 |
|  | Simple mode | 719 | 0.88(0.51-1.54) | -0.12 | 0.28 | 0.66 |
|  | Weighted mode | 719 | 0.94(0.69-1.29) | -0.06 | 0.16 | 0.71 |
| MPV | IVW | 785 | 1.08(0.99-1.17) | 0.07 | 0.04 | 0.09 |
|  | WM | 785 | 1.09(0.95-1.25) | 0.09 | 0.07 | 0.21 |
|  | MR Egger | 785 | 1.00(0.87-1.15) | 0.00 | 0.07 | 0.96 |
|  | Simple mode | 785 | 1.16(0.82-1.64) | 0.15 | 0.18 | 0.41 |
|  | Weighted mode | 785 | 1.08(0.92-1.28) | 0.08 | 0.08 | 0.34 |
| RBC | IVW | 554 | 0.95(0.81-1.11) | -0.05 | 0.08 | 0.50 |
|  | WM | 554 | 0.95(0.73-1.24) | -0.05 | 0.13 | 0.72 |
|  | MR Egger | 554 | 0.99(0.71-1.37) | -0.01 | 0.17 | 0.95 |
|  | Simple mode | 554 | 0.88(0.41-1.90) | -0.12 | 0.39 | 0.75 |
|  | Weighted mode | 554 | 1.08(0.66-1.79) | 0.08 | 0.26 | 0.76 |
| RDW | IVW | 16 | 0.73(0,45-1.19) | -0.31 | 0.25 | 0.20 |
|  | WM | 16 | 0.68(0.36-1.28) | -0.39 | 0.33 | 0.23 |
|  | MR Egger | 16 | 1.58(0.39-6.33) | 0.46 | 0.71 | 0.53 |
|  | Simple mode | 16 | 0.75(0.28-2.00) | -0.29 | 0.50 | 0.57 |
|  | Weighted mode | 16 | 0.68(0.31-1.52) | -0.38 | 0.41 | 0.36 |
| WBC | IVW | 465 | 1.04(0.89-1.22) | 0.04 | 0.08 | 0.64 |
|  | WM | 465 | 1.22(0.93-1.60) | 0.20 | 0.14 | 0.16 |
|  | MR Egger | 465 | 1.09(0.76-1.57) | 0.09 | 0.18 | 0.63 |
|  | Simple mode | 465 | 1.36(0.64-2.92) | 0.31 | 0.39 | 0.42 |
|  | Weighted mode | 465 | 1.32(0.85-2.06) | 0.28 | 0.23 | 0.22 |
| NETU | IVW | 384 | 1.08(0.91-1.28) | 0.07 | 0.09 | 0.40 |
|  | WM | 384 | 1.16(0.85-1.58) | 0.15 | 0.16 | 0.35 |
|  | MR Egger | 384 | 1.03(0.71-1.51) | 0.03 | 0.19 | 0.87 |
|  | Simple mode | 384 | 1.25(0.54-2.87) | 0.22 | 0.42 | 0.60 |
|  | Weighted mode | 384 | 1.29(0.84-1.97) | 0.25 | 0.22 | 0.25 |
| NEUT% | IVW | 349 | 1.10(0.91-1.32) | 0.09 | 0.09 | 0.32 |
|  | WM | 349 | 1.25(0.94-1.66) | 0.23 | 0.14 | 0.12 |
|  | MR Egger | 349 | 1.33(0.86-2.07) | 0.29 | 0.23 | 0.20 |
|  | Simple mode | 349 | 0.91(0.39-2.15) | -0.09 | 0.44 | 0.83 |
|  | Weighted mode | 349 | 1.63(0.97-2.73) | 0.49 | 0.26 | 0.07 |
| LYM | IVW | 489 | 0.88(0.76-1.02) | -0.13 | 0.07 | 0.08 |
|  | WM | 489 | 0.79(0.62-0.99) | -0.24 | 0.12 | 0.04 |
|  | MR Egger | 489 | 0.91(0.65-1.27) | -0.09 | 0.17 | 0.58 |
|  | Simple mode | 489 | 0.72(0.36-1.43) | -0.33 | 0.35 | 0.35 |
|  | Weighted mode | 489 | 0.78(0.51-1.21) | -0.25 | 0.22 | 0.27 |
| LYM% | IVW | 395 | 0.90(0.75-1.07) | -0.11 | 0.09 | 0.23 |
|  | WM | 395 | 0.87(0.64-1.19) | -0.14 | 0.16 | 0.39 |
|  | MR Egger | 395 | 0.84(0.55-1.29) | -0.17 | 0.22 | 0.43 |
|  | Simple mode | 395 | 0.74(0.30-1.84) | -0.30 | 0.47 | 0.51 |
|  | Weighted mode | 395 | 0.72(0.42-1.22) | -0.33 | 0.27 | 0.22 |

b, regression coefficient; IVW, inverse variance weighted; LYM, lymphocyte cell count; LYM%, lymphocyte cell count percentage; MPV, Mean platelet (thrombocyte) volume; NEUT, Neutrophil; NEUT%, Neutrophil percentage; N SNP, number of single nucleotide polymorphisms; SE: standard error; OR, odds ratio; PDW, Platelet distribution width; PDW_1_, Data from GWAS ID ukb-d-30110_irnt; PDW_2,_ Data from GWAS ID ebi-a-GCST004616; PLC, Platelet count; RBC, Red blood cell (erythrocyte) count; RDW, Red blood cell (erythrocyte) distribution width; WBC, White blood cell (leukocyte) count; WM, weighted median; UVMR, Univariable Mendelian randomization. ^a^ Results from 2-sample UVMR analysis; estimated associations reported as OR of outcome per unit increase in log odds of 28-day mortality in sepsis. ^b^ All relevant SNPs were identified in a GWAS as having reached a selection threshold of P<0.05×10^-8^ and pruned at linkage disequilibrium R^2^<0.001.

**Table S4. The detailed UVMR results of Casual effect of 28-day mortality in sepsis on PDW^a,b^.**

| Exposure | Methods | N SNP | OR (95%CI) | b | SE | P value |
| --- | --- | --- | --- | --- | --- | --- |
| 28-day mortality in sepsis | IVW | 27 | 1.00(1.00-1.01) | 0 | 0 | 0.29 |
|  | WM | 27 | 1.00(0.99-1.01) | 0 | 0 | 0.82 |
|  | MR Egger | 27 | 1.00(1.00-1.02) | 0.01 | 0.01 | 0.19 |
|  | Simple mode | 27 | 1.00 (0.98-1.01) | 0 | 0.01 | 0.83 |
|  | Weighted mode | 27 | 1.00(0.98-1.01) | 0 | 0.01 | 0.86 |

b, regression coefficient; IVW, inverse variance weighted; WM, weighted median; N SNP, number of single nucleotide polymorphisms; SE: standard error; OR, odds ratio; PDW, Platelet distribution width; PDW, Data from GWAS ID ukb-d-30110_irnt; UVMR, Univariable Mendelian randomization.

^a^ Results from 2-sample UVMR analysis; estimated associations reported as OR of outcome per unit increase in log odds of PDW. ^b^ All relevant SNPs were identified in a GWAS as having reached a selection threshold of P<0.05×10^-8^ and pruned at linkage disequilibrium R^2^<0.001.

**
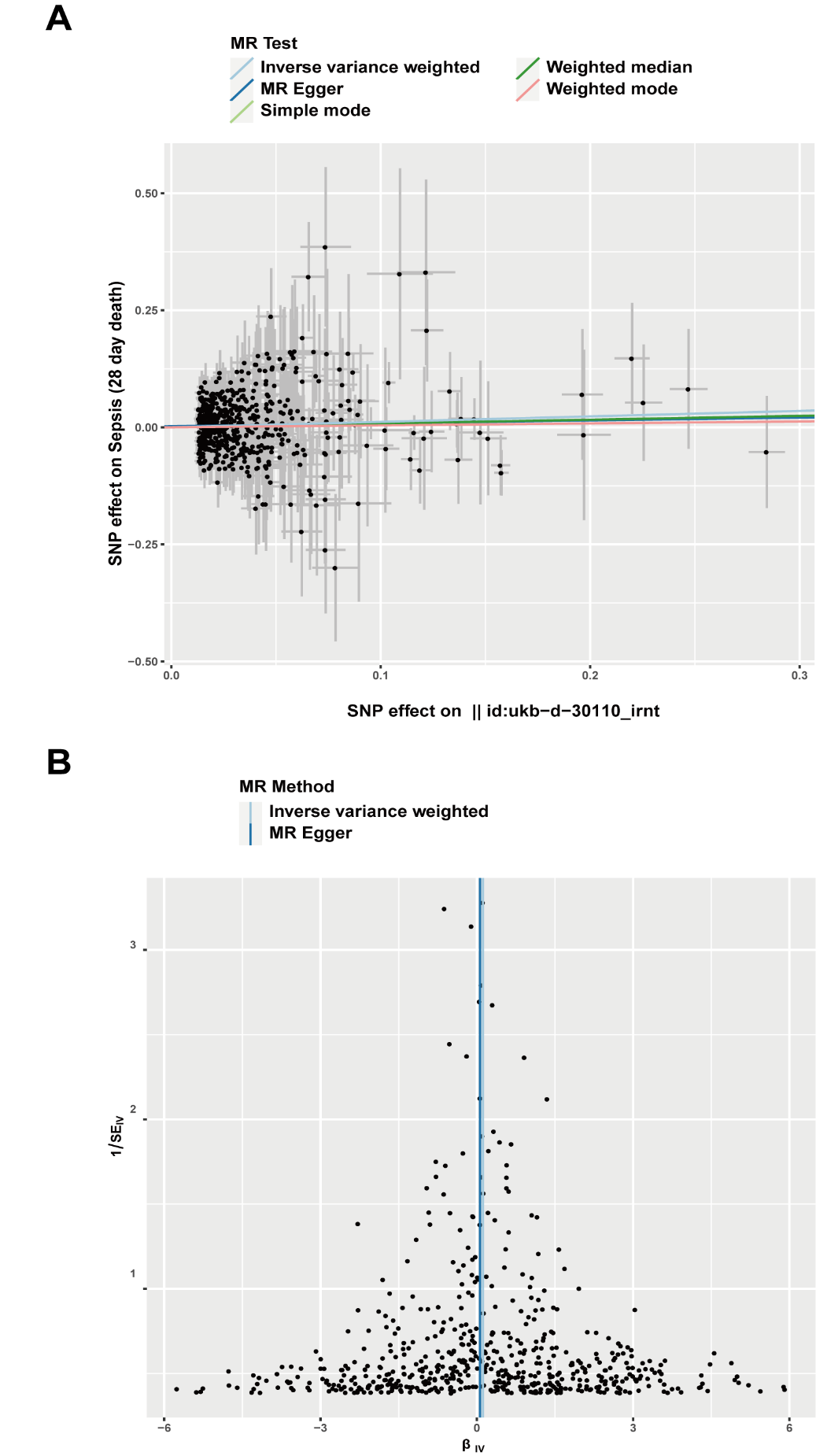
****Figure S1 |** **scatter plot (A) and funnel plot (B) of the causal effect of PDW on 28-day mortality in sepsis risk after removing SNPs with potential pleiotropy.**
